# Integrating serum pharmacochemistry and network pharmacology to reveal the active constituents and mechanism of Corydalis Rhizoma in treating Alzheimer’s disease

**DOI:** 10.3389/fnagi.2023.1285549

**Published:** 2023-11-22

**Authors:** Yan Lyu, Yu Wang, Jianyou Guo, Yuqing Wang, Yifan Lu, Zhuangzhuang Hao, Tingyue Jiang, Wenxin Fan, Yihua Li, Jinli Shi

**Affiliations:** ^1^School of Chinese Materia Medica, Beijing University of Chinese Medicine, Beijing, China; ^2^CAS Key Laboratory of Mental Health, Institute of Psychology, Chinese Academy of Sciences, Beijing, China

**Keywords:** Corydalis Rhizoma, Alzheimer’s disease, serum pharmacochemistry, network pharmacological, neuroinflammation, polarization type of BV2, JAK2/STAT3

## Abstract

**Background:**

Alzheimer’s disease (AD) is a multifactorial neurodegenerative condition. The search for multi-target traditional Chinese medicines or ingredients for treating AD has attracted much attention. Corydalis rhizome (CR) is a traditional Chinese medicine. Its main components are alkaloids, which have therapeutic effects that can potentially be used for treating AD. However, no systematic study has been conducted to explore the anti-AD efficacy of CR, as well as its active compounds and mechanisms of action.

**Objective:**

The present study aimed to clarify CR’s active constituents and its pharmacological mechanisms in treating AD.

**Methods:**

A D-galactose & scopolamine hydrobromide-induced AD mouse model was used and CR was administered orally. The prototypical alkaloid components were identified in the serum. The core components, key targets, and possible mechanisms of action of these alkaloids were revealed through network pharmacology. Molecular docking of the key target was performed. Finally, the mechanism was validated by lipopolysaccharide (LPS)-induced activation of BV2 microglia.

**Results:**

The results showed that CR improved anxiety-like behavior, spatial and non-spatial recognition, and memory capacity in AD mice. It also achieved synergistic AD treatment by modulating neurotransmitter levels, anti-neuroinflammation, and anti-oxidative stress. The core components that enhance CR’s efficacy in treating AD are protoberberine-type alkaloids. The CR may induce the polarization of LPS-activated BV2 microglia from phenotype M1 to M2. This is partially achieved by modulating the IL-6/JAK2/STAT3 signaling pathway, which could be the mechanism by which CR treats AD through anti-inflammation.

**Conclusion:**

The present study provided a theoretical and experimental basis for the clinical application of CR in treating AD. It also provides information that aids the secondary development, and precise clinical use of CR.

## 1 Introduction

Alzheimer’s disease (AD) is an insidious progressive neurodegenerative condition. Cholinergic neurotransmitter deficiency, neuroinflammation, oxidative stress, and deposition of Aβ are vital in the development of AD (Lane et al., 2018; DeTure and Dickson, 2019). Massive efforts were devoted to discovering anti-AD therapies based on these pathogenic factors. However, the “one drug for one target” strategy has been dominant, and the progress in treating AD has been limited and disappointing (Huang et al., 2020; Cummings et al., 2021). Due to the complex pathogenesis of AD, single-target therapeutic strategies may have limitations, and the search for multi-target, multi-pathway therapies for AD has become an important alternative (Sang et al., 2022).

Chinese medicine or active ingredients have attracted much attention due to their unique multi-target and multi-pathway therapeutic effects (Singh et al., 2020). Corydalis rhizome (CR) is the dried tuber of *Corydalis yanhusuo* W. T. Wang, which belongs to the family Papaveraceae. CR has analgesic, hypnotic, sedative, and anti-inflammatory effects. It is one of the traditional Chinese medicines and is also used in Danish folklore for memory dysfunction (Adsersen et al., 2006; Novak et al., 2012; Liang et al., 2020). Modern pharmacological studies showed that the total alkaloids of CR, tetrahydropalmatine, palmatine, berberine, dehydrocorydaline, corydalis polysaccharide, and Yuan-Hu Zhi Tong Prescription could inhibit AChE (Xiao et al., 2011; Wang et al., 2018), protect neurons from the influence of some harmful factors such as neuroinflammation (Liu et al., 2021; Wen et al., 2022), oxidative stress (Long et al., 2019; Jia et al., 2021), excitotoxicity (Lin et al., 2022), Aβ aggregation, and tau protein aggregation (Cai et al., 2018; Iyaswamy et al., 2020; Du et al., 2022). However, the anti-AD efficacy of CR has not been systematically studied, along with the therapeutic effects, active substances, and possible mechanisms of the rhizome.

Serum pharmacochemistry aims to analyze human or animal blood components after the oral administration of Chinese medicine. It is based on modern separation and analysis techniques such as chromatography and mass spectrometry, Serum pharmacochemistry is an effective way to clarify the potential pharmacodynamic substances of Chinese medicines (Han et al., 2020). pharmacology and serum pharmacochemistry can complement each other to predict pharmacological mechanisms by determining the active components that act directly *in vivo* (Tang et al., 2022; Liang et al., 2023).

The pathogenesis of AD is complex due to the interaction of multiple factors. So, no pathological model could fully reproduce or simulate all clinicopathological changes of AD. The AD composite model is a combination of two or more AD modeling methods that can simulate the pathological features of AD to a greater extent from multiple perspectives and is increasingly popular (Xia et al., 2023). D-galactose (D-gal), an aldohexose, is widely used to induce age-related diseases, including AD (Azman and Zakaria, 2019). Continuous subcutaneous injection of D-gal to mice can establish a subacute aging model, which is similar to the natural aging model, and result in pathological changes such as memory dysfunction, neuroinflammation, oxidative stress, deposition of Aβ in the hippocampus, and reduction of neurons (Ali et al., 2021; Kandeda et al., 2022; Anand et al., 2023). Scopolamine hydrobromide (Scop) is a competitive muscarinic-type acetylcholine receptor antagonist, which is widely used for preliminarily screening intelligence-promoting drugs and drugs against AD. The intraperitoneal injection of Scop may block the binding sites of the acetylcholine receptor in the cerebral cortex, septal regions, and hippocampus. This causes cholinergic neural signaling deficits and cognitive decline, which resembles the pathological process of AD (More et al., 2016; Chen and Yeong, 2020; Ni et al., 2023). The combination of D-gal and Scop modeling can complement each other.

**Figure 1:**
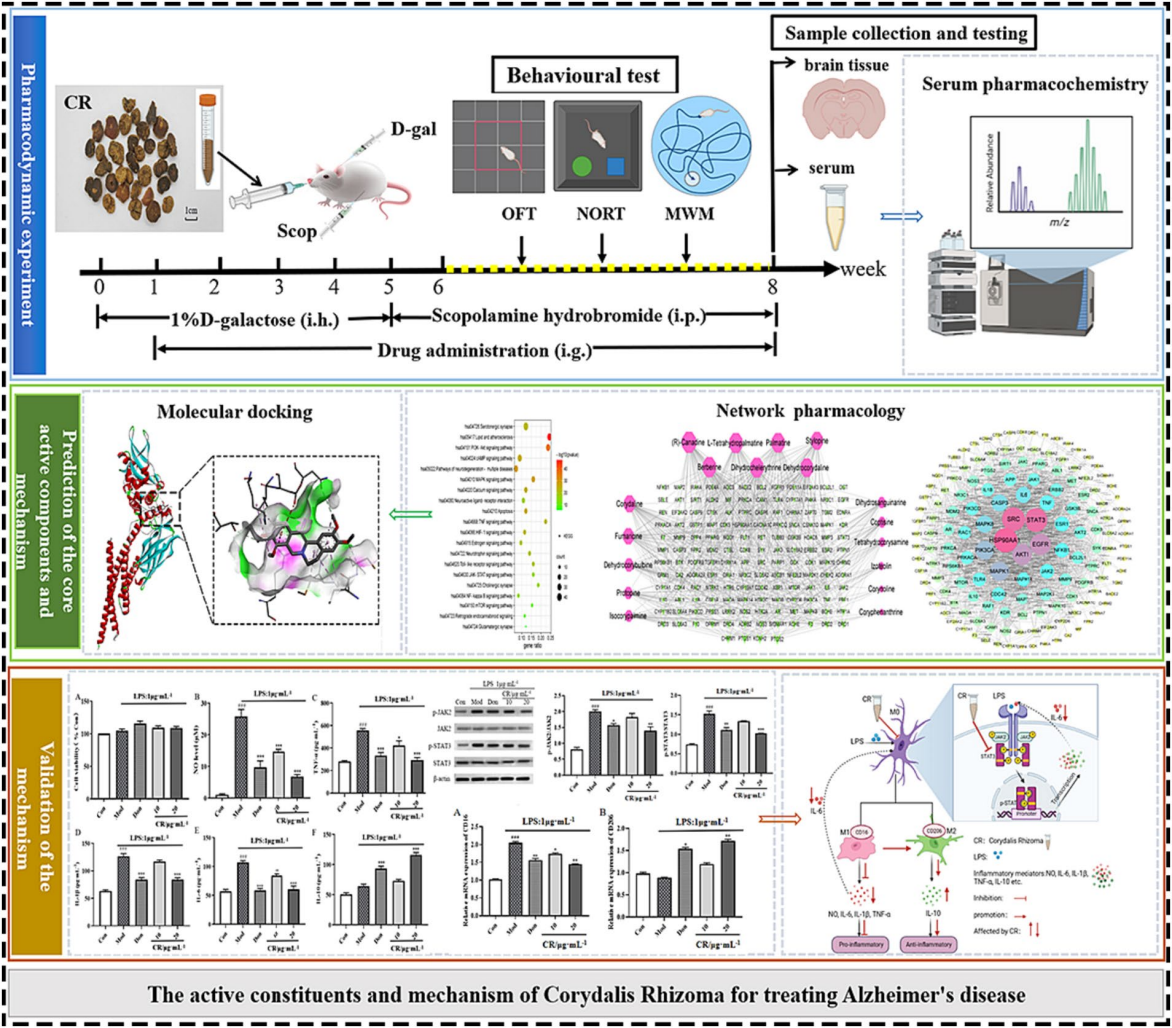
Schematic diagram of an integrated systems pharmacology-based framework for deciphering the pharmacodynamic substance and mechanism of CR against AD.

In this study, the AD mouse model induced by D-gal and Scop (D-gal & Scop) was adopted, which multiples the simulated pathological and clinical features of AD. The efficacy of CR in treating AD was evaluated using classical behavioral experiments, together with the measurements of biochemical indexes of the brain. Then, serum pharmacochemistry was used to identify the blood-entry alkaloid prototypical components of CR as candidate pharmacodynamic components. At the same time, the core active alkaloid components, key targets, and possible mechanisms of CR for AD treatment were screened using network pharmacology. This was followed by molecular docking of the core active components and key targets. Finally, LPS-induced BV2 and an *in vitro* neuroinflammation model were established to validate the key targets and important pathways (Figure 1).

## 2 Materials and methods

### 2.1 Drugs and reagents

L-Tetrahydropalmatine (PCS0603), Palmatine (PCS0470), Dehydrocorydaline (PCS0968), (R)-Canadine (PCS0136), Protopine (PCS0963), D-Corydaline (PCS0604) were purchased from Chengdu Herb Substance Technology Co., Ltd.; Columbamine (RFS-F02002003011) was purchased from Chengdu Ruifenshi Biotechnology Co., Ltd.; Glaucine (HR11395W2), Coptisine (MUST-21082002) were purchased from Chengdu Must Bio-Technology Co., Ltd.; Berberine (C12299673) was purchased from Shanghai Macklin Biochemical Co., Ltd. Donepezil hydrochloride (Zhejiang Huahai Pharmaceutical Co., Ltd., 0000013745) was used as the positive drug. D-galactose (Sigma-Aldrich, WXBD0429V) and scopolamine hydrobromide (Maklin, C12740415) were used to induce AD mouse models. BV2 cells were induced by lipopolysaccharide (LPS, Sigma, from *Escherichia coli* 055: B5) to form a neuroinflammatory model *in vitro*. ELISA kits for ACh, AChE, IL-1β, IL-6, COX-2, TNF-α, iNOS, NO, MDA, SOD and GSH-Px were all obtained from Jiangsu MeimianBiotechnology Co., Ltd. (MM-0520 M1, MM-0674 M1, MM-0040 M1, MM-0163 M1, MM-0356 M1, MM-0132 M1, MM-0454 M1, MM-0658 M1, MM-0897 M1, MM-0389 M1, MM-44117 M1). NO kit and CCK8 kit were purchased from Beijing Biorigin Biotechnology Co., Ltd. Actin, JAK2, p-JAK2, STAT3, p-STAT3, NF-κB, p-NF-κB, iNOS were obtained from Jiangsu Affinity Biosciences Co., Ltd. (AF7018, AF6022, AF3024, AF6294, AF3293, AF8005, AF3219, AF0199). The BCA protein assay kit was obtained from Beijing Medical Discovery Leader Biotechnology Co., Ltd. (MD913053).

### 2.2 Preparation of the Corydalis Rhizoma (CR) extract

The CR samples were obtained from Shandong Dexintang Pharmaceutical Co., Ltd and identified by Prof. Jin-Li Shi from the Department of Authentication of Chinese Medicines at Beijing University of Chinese Medicine.

The CR samples were crushed and passed through a 60-mesh sieve. About 100 g of the powdered sample was accurately weighed into a flat-bottomed flask, prior to the addition of 800 mL distilled water (1:8, *w*/*v*). Then, the sample was sonicated at 35°C for 30 min (800 W). After filtration, the residue underwent reflux extraction using 70% ethanol solution (1:8, *w*/*v*) for 2 h, with two repetitions. The filtrate was collected and dried under reduced pressure at 45°C. The CR extract was obtained.

A precisely weighted CR extract (100 mg) was disposed into a 15 mL centrifuge tube. After adding 10 mL of 50% methanol solution into the centrifuge tube, the extract was sonicated for 30 min. The resulting solution was centrifuged at 14,000 r/min for 10 min at 4°C to make the sample solution, which was then filtered through a 0.22 μm membrane before injection. The filtrate (5 μL) was injected into the UPLC-Q-TOF-MS for analysis.

### 2.3 Animals and treatment

ICR male mice, 5-week-old weighing (20 ± 2) g, were purchased from Beijing SPF Biotechnology Co., Ltd. (Certificate No.: SCXK-2019-0010) and kept in a standardized environment with a 12-h light/dark cycle (light on 7: 00–19: 00) with (22 ± 2) °C room temperature and (60 ± 5)% relative humidity. All animals received a standard diet and water *ad libitum*. All experiments involving animals were performed per the National Institutes of Health’s Guide for the Care and Use of Laboratory Animals and were approved by the Animal Care and Use Committee of Beijing University of Chinese Medicine (No. BUCM-4-2021092601-3090).

ICR mice were randomly divided into six groups: control group (Con); AD model group (Mod); donepezil hydrochloride group (Don, 1.517 mg/kg, positive drug control group); CR low-dose group (CR-L, 0.7583 g/kg); CR medium-dose group (CR-M, 1.517 g/kg); and CR high-dose group (CR-H, 3.033 g/kg).

The AD model was established according to the reference method (Muhammad et al., 2019; Fasina et al., 2022). The mice, except for those in the control group, were subcutaneously injected with 0.1% D-gal solution (D-gal dissolved with normal saline) on the back of the neck, at a dose of 150 mg/kg body weight once a day for 5 weeks. From week 6, all mice (except those in the control group) were intraperitoneally injected with Scop at 3 mg/kg body weight once a day for 3 weeks. Meanwhile, those in the control group were given the same volume of normal saline, instead of D-gal and Scop. From weeks 2 to 8, the mice in the therapy group were given the Don or CR extract by gavage. The same volume of normal saline was simultaneously administered to the mice in the control and model groups. After 42 days of administering the treatments, the behavioral test was performed. The drug was administered by gavage 1 h before the behavioral test and intraperitoneally 30 min prior. The scheme of the experimental design is shown in Figure 1.

### 2.4 Behavioral test

#### 2.4.1 Open field test

The open field test (OFT) was used to evaluate the mice’s autonomous exploration behavior and motor ability (Wu et al., 2020). The experimental device is a square-shaped box with a floor (50 × 50 cm) and a boundary wall (50 cm high). We divided the surface of the open field into 25 equal squares. The central area had nine squares in the middle of the open field. Each mouse was placed in the same edge zone of the open field and allowed to move freely for 5 min. The speed at which the mice moved and the frequency at which they entered the central area were recorded.

#### 2.4.2 Novel object recognition test

The Novel object recognition test (NORT) is widely used for assessing hippocampus-dependent recognition memory and is based on the animals’ tendency to spend more time investigating a novel object than a familiar one (Lueptow, 2017). The mouse was placed in the open field (50 × 50 × 50 cm) for adaptation 2 days before the test. During the training phase, the mouse was given 5 min to freely explore two objects of the same shape, color, and size. An hour after the end of the training phase, one of the objects in the open field was replaced with a new one (different in shape and color compared to the old one). Then, the mouse was allowed to freely explore the new and old objects in the open field for 5 min. After removing each mouse, the open field and the objects were carefully cleaned with 75% ethanol. The recognition index (T) a used to evaluate the recognition memory ability of the tested animals. The recognition index was regarded as the ratio of the time spent exploring the novel object over the total time inspecting both the new and old objects (Salem et al., 2021). New object recognition memory was demonstrated when the T was higher than the random level (50%). The higher the T, the stronger the new object recognition memory (Lueptow, 2017).

#### 2.4.3 Morris water maze test

The spatial learning and memory of mice were assessed using the Morris water maze test (MWM) (Wang Z. et al., 2023). The swimming maze was a circular pool with black walls (120 cm in diameter and 50 cm in height). The maze was filled with water (22 ~ 24°C) to a depth of 30 cm. Four points were designated on the rim of the tank, thus dividing the pool into four quadrants (I, II, III, and IV). The fifth quadrant was a black submerged platform (6.5 cm in diameter and 29 cm in height) located at the center of the IV quadrant, 1 cm beneath the water’s surface. The water was made opaque by adding black ink to render the platform invisible. The training phase continued for 4 days. The mice were released into the pool from different quadrants (I, II, III, or IV) and allowed to swim for 60 s. The escape latency was recorded when the mouse found the hidden platform. If not, the mouse was artificially guided to the platform and stayed for 15 s. In such cases, the escape latency was recorded as 60 s. At the spatial probe phase (day 5), the hidden platform was removed, and mice were released from the II quadrant pool and allowed to swim for 60 s. The number of platform crossings was recorded.

### 2.5 Nissl staining

The brain tissue was immersed in 4% paraformaldehyde solution and fixed for 24 h, then, according to standard procedures, dehydrated, embedded, sliced 4 μm, and stained with Nissl staining solution. The sections were observed under optical microscopes (ECLIPSETS 100, Nikon).

### 2.6 ELISA

The levels of AChE, IL-1β, IL-6, COX-2, TNF-α, iNOS, NO, MDA, SOD, and GSH-Px in the brain tissue were measured by ELISA. The experiments were performed strictly according to the kit instructions.

### 2.7 Preparation of serum samples

After the behavioral test, three mice were randomly selected from the AD model and the CR high-dose groups, respectively. In the CR high-dose group, the CR extract was administered by gavage at a 3.033 g/kg dose for three consecutive days. The AD model group was given the same volume of saline. The mice fasted for 12 h and were allowed to freely access water before the last administration session. Approximately 500 μL whole blood was collected an hour after the final administration procedure. The serum was prepared by centrifuging the whole blood at 3,500 rpm at 4°C for 10 min, after standing for 30 min at room temperature. The whole blood was stored at −80°C until the analysis was done. Serum samples from the same group were mixed (100 μL for each mouse, 300 μL total) and added to 500 μL of 50% acetonitrile to precipitate the protein. The mixture was then vortexed for 3 min and centrifuged at 14,000 rpm and 4°C for 10 min. The supernatant was then transferred and dried using nitrogen gas at 40°C. The residues were redissolved in 100 μL of 50% acetonitrile. Filtration was done using a 0.22 μm membrane before injection.

### 2.8 UPLC-Q-TOF-MS conditions

The chemical constituents of the CR extract were analyzed using an ACQUITY UPLC I Class system (Waters, United States) equipped with Synapt G2-Si Qtof mass spectrometry (Waters, United States). Chromatographic separation was performed on the ACQUITY UPLC HSS T3 column (2.1 × 100 mm, 1.8 μm) at 40°C with a 0.3 mL/min flow rate. The mobile phase consisted of 0.1% (v/v) aqueous formic acid (A) and acetonitrile (B) with gradient elution as follows: 10% B at 0–10 min; 10–15% B at 10–15 min; 15–20% B at 15–25 min; 20–40% B at 25–35 min; 40–70% B at 35–45 min; 70% B at 45–47 min; 70–10% B at 47–49 min; 10% B at 49–50 min. The injection volume was 5 μL. The MS conditions were set to scan range m/z 150–1,500 Da at positive ion mode, and the capillary voltage was 2.5 kV (−). The ESI electrospray ionization source was used, and the ion source temperature was 125°C. The cone hole voltage was 40 V and the collision energy was 10–50 V. Desolvation gas and cone gas flow rates were 800 L/H and 50 L/H, respectively. The desolvation temperature was 500°C.

Data were collected using the Masslynx v4.2 software (Waters, Milford, United States) in the positive ion mode. The UNIFI™ v1.8 software (Waters, Milford, United States) was used for processing the data for peak picking and alignment. According to molecular ion and isotope peaks, possible molecular formulas were fitted using the Masslynx v4.2 software and matched with mzVault and mzCloud databases (target mass match tolerance <10 ppm, matching degree of MS/MS fragmentation >50%), to infer the possible chemical composition. The chemical composition was identified with the relative retention time and MS/MS fragmentation of reference substances, literature, and databases.

### 2.9 Network pharmacology

#### 2.9.1 Screening of candidate bioactive components of CR

Based on the results of serum pharmacochemistry and Traditional Chinese Medicine Systems Pharmacology,^1^ the CR components absorbed into the blood of mice with oral bioavailability (OB) ≥30% and drug-likeness (DL) ≥0.18 were selected as candidate bioactive compounds.

#### 2.9.2 Acquisition of potential targets of anti-AD

The targets associated with candidate compounds were collected through Traditional Chinese Medicine Systems Pharmacology (see text footnote 1), SwissTargetPrediction,^2^ and ETCM^3^ databases. All targets were combined, and duplicates were removed. The related AD targets were searched using “Alzheimer’s disease” as the keyword from Gene Cards,^4^ TTD,^5^ and OMIM^6^ databases. The targets of the Gene Cards database were selected with a relevance score greater than 10.0. The UniProt database corrected the information of target proteins and genes, and “*Homo sapiens*” targets were screened out. The Venn diagram matched the overlapping targets between the potential therapeutic targets of CR and AD disease targets.

#### 2.9.3 Protein–protein interaction (PPI) network

The potential targets for anti-AD were entered into the STRING database^7^ to establish the PPI network. The species setting was limited to “*Homo sapiens*” and the cut-off value was 0.7. The key targets were obtained using the Cytoscape 3.9.1 software.

#### 2.9.4 Compound-target network

The candidate bioactive components and potential targets were imported into Cytoscape 3.9.1 to construct a compound-target network. The critical bioactive components were screened.

#### 2.9.5 Gene ontology and pathway enrichment analysis

To further elucidate the anti-AD mechanism of CR, the potential targets for anti-AD were imported into the Metascape database^8^ for gene ontology (GO) and the Kyoto Encyclopedia of genes and genomes (KEGG) enrichment analysis. The threshold was set at lower than 0.01.

#### 2.9.6 Molecular docking

Molecular docking was further performed to verify the binding ability of the key targets and components. The core pharmacodynamic constituents were selected as ligands. The SDF files of the corresponding ligands were obtained from the PubChem database and converted into mol2 files using the GaussView software. The STAT3 (PDB ID: 6NJS) protein was obtained by the Protein Data Bank database.^9^ Using the PyMOL 2.3.4 software, water and ligands were removed from the receptor protein. Hydrogenation and charge calculation of the selected receptor proteins were first performed using AutoDock Vina, after which docking of small ligand molecules and receptor proteins followed. The appropriate box center coordinates and box size were determined before docking. The Discovery studio visualizer was also used to draw the conformation with the lowest docking binding energy.

### 2.10 Cellular experimental verification

#### 2.10.1 Cell culture and treatment

The BV2 immortalized microglial cell line was obtained from BeNa Culture Biotechnology Co., Ltd. and cultured in DMEM medium (with 10% fetal bovine serum, 1% penicillin-streptozotocin) at 37°C with 5% CO_2_. The control (Con), LPS (1 μg·mL^−1^) (Mod), LPS + Don (40 μM) (Don), and LPS + CR (10 and 20 μg·mL^−1^) groups were set up. BV2 cells in the logarithmic growth phase were collected and inoculated on culture dishes for 24 h. After removing the medium, Don and CR solutions were added at final concentrations of 40 μM, 10 μg·mL^−1^, and 20 μg·mL^−1^, respectively. After further incubation for 30 min, LPS (final concentration of 1 μg·mL^−1^) was added and placed in the incubator for 24 h.

#### 2.10.2 CCK-8 assay for cell viability

The cell density was adjusted, before inoculating the BV2 cells in 96-well plates. The culture was operated according to the experimental grouping in Section 2.10.1. After 24 h, the CCK-8 method was used to detect the absorbance of drug-activated cells at 450 nm after 24 h using.

#### 2.10.3 Griess method for NO level

The cell supernatant (50 μL) from each group was taken and placed into a 96-well plate. Five duplicate wells were used per group. Sequentially, 50 μL of Griess reagents I and II were added prior to mixing well. The absorbance (OD) value for each well at 540 nm was detected using a microplate reader, and the NO content of the supernatant was calculated based on the standard curve.

#### 2.10.4 ELISA

The supernatants of the collected cells from each group were placed in a −80°C fridge, with 5 replicate wells in each group. The levels of IL-1β, IL-6, IL-10, and TNF-α were measured in strict accordance with the ELISA kit instructions.

#### 2.10.5 Western blotting

Groups of cells were collected, and a pre-cooled RIPA lysis buffer that contained protease inhibitors was added to them. The cells were broken through ultrasonication in an ice bath. They were then centrifuged, and the supernatant was collected to determine the concentration of proteins using a BCA kit. An equal amount of protein sample was separated using 10% SDS-PAGE electrophoresis before being transferred to the PVDF membrane. The membranes were blocked with 5% skimmed milk for an hour at room temperature and then incubated with primary antibodies, overnight at 4°C. The dilution rates of the primary antibodies were as follows: β-actin = 1:2,000; JAK2 = 1:1,000; p-JAK2 = 1:1,000; STAT3 = 1:1,000; p-STAT3 = 1:1,000; NF-κB = 1:1,000; p-NF-κB = 1:1,000; iNOS = 1:1,000. The membranes were then incubated with horseradish peroxidase (HRP)-conjugated secondary antibodies (1:5,000; goat anti-mouse) for an hour, at room temperature. Images were acquired using a gel imaging analysis system (ChemiDoc MP, United States), and the protein bands were analyzed using the Image J software.

#### 2.10.6 RT-qPCR

The total RNA of BV2 cells was extracted using the Trizol method. The RNA was then reverse transcribed to obtain the corresponding cDNA, according to the instructions on the RevertAid First Strand cDNA Synthesis Kit. Then, amplification was conducted using quantitative Real-time PCR (RT-qPCR) following the instructions on the ServicebioRT Enzyme Mix kit. The primers are described in Supplementary Table S1.

### 2.11 Statistical analysis

Behavioral data were analyzed by EthoVision XT 9 software. GraphPad Prism 8.0 software was used for graphing. SPSS 20.0 software was used to perform statistical analysis. The results are expressed as $\bar{X}$ ± SEM. For data meeting normal distribution and homogeneity of variance, *t*-test was used for comparison between groups, and one-way analysis of variance (ANOVA) was used for comparison between multiple groups; if homogeneity of variance was not met, *t*’ test and Welch’s ANOVA analysis were used. Otherwise non-parametric tests will be used. *p* < 0.05 was considered significant.

## 3 Results

### 3.1 A preliminary study on the efficacy of CR in the treatment of AD

#### 3.1.1 Effects of CR on the behavior of AD mice

##### 3.1.1.1 Open field test (OFT)

**Figure 2:**
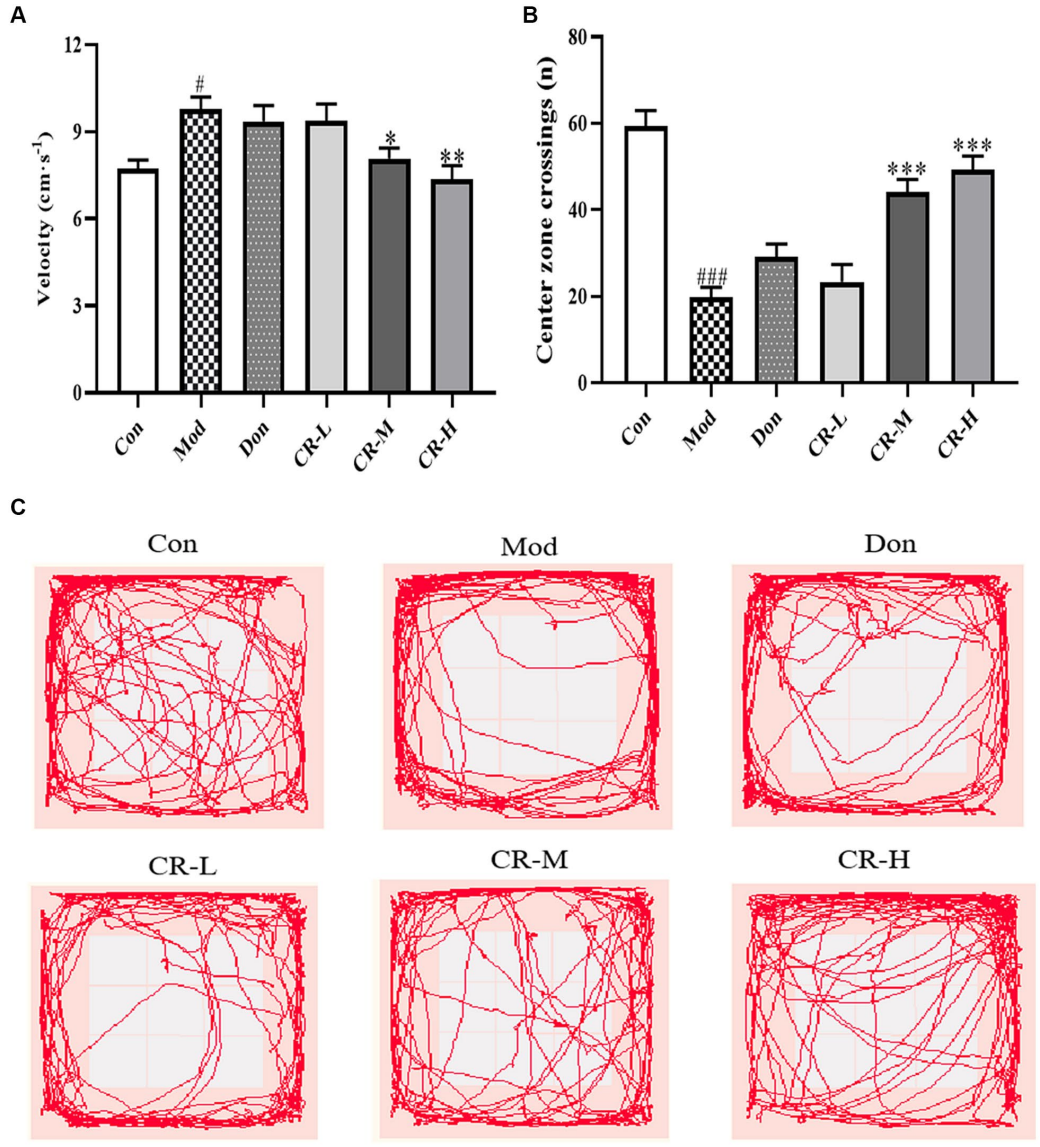
Effects of CR on the velocity **(A)**, number of crossings of the central zone **(B)** and tracks **(C)** of mice in the OFT. The data were presented as mean ± SEM, *n* = 9 ~ 11; ^#^*p* < 0.05, ^##^*p* < 0.01, ^###^*p* < 0.001 vs. the Con group; ^*^p < 0.05, ^**^p < 0.01, ^***^p < 0.001, vs. the Mod group.

The results of the OFT are shown in Figure 2 (Supplementary Table S2). Compared to the control mice (Con), the AD model mice (Mod) showed abnormal autonomic activity in the open field, with an increase in their velocity (*p* < 0.05) and a significant decrease in the number of crossings through the central zone (*p* < 0.001). Although the modeling method did not affect the AD mice’s mobility, they showed mild anxiety-like behavior. There was no significant difference in the velocity and number of times for crossing the central zone in the donepezil hydrochloride group mice (Don) compared to the AD model mice. The velocity in the CR low-dose group (CR-L) mice decreased, though no statistical significance was observed compared to the AD model mice. The CR medium-dose (CR-M) and CR high-dose (CR-H) groups exhibited differences in the extent to which velocity of movement was reduced (*p* < 0.05, *p* < 0.01). The frequency of crossing the central zone significantly increased in the CR-M and CR-H groups (*p* < 0.001) but not in the CR-L group. This indicated that CR improved the anxiety-like behavior in AD mice in a dose-dependent manner.

##### 3.1.1.2 Novel object recognition test (NORT)

**Figure 3:**
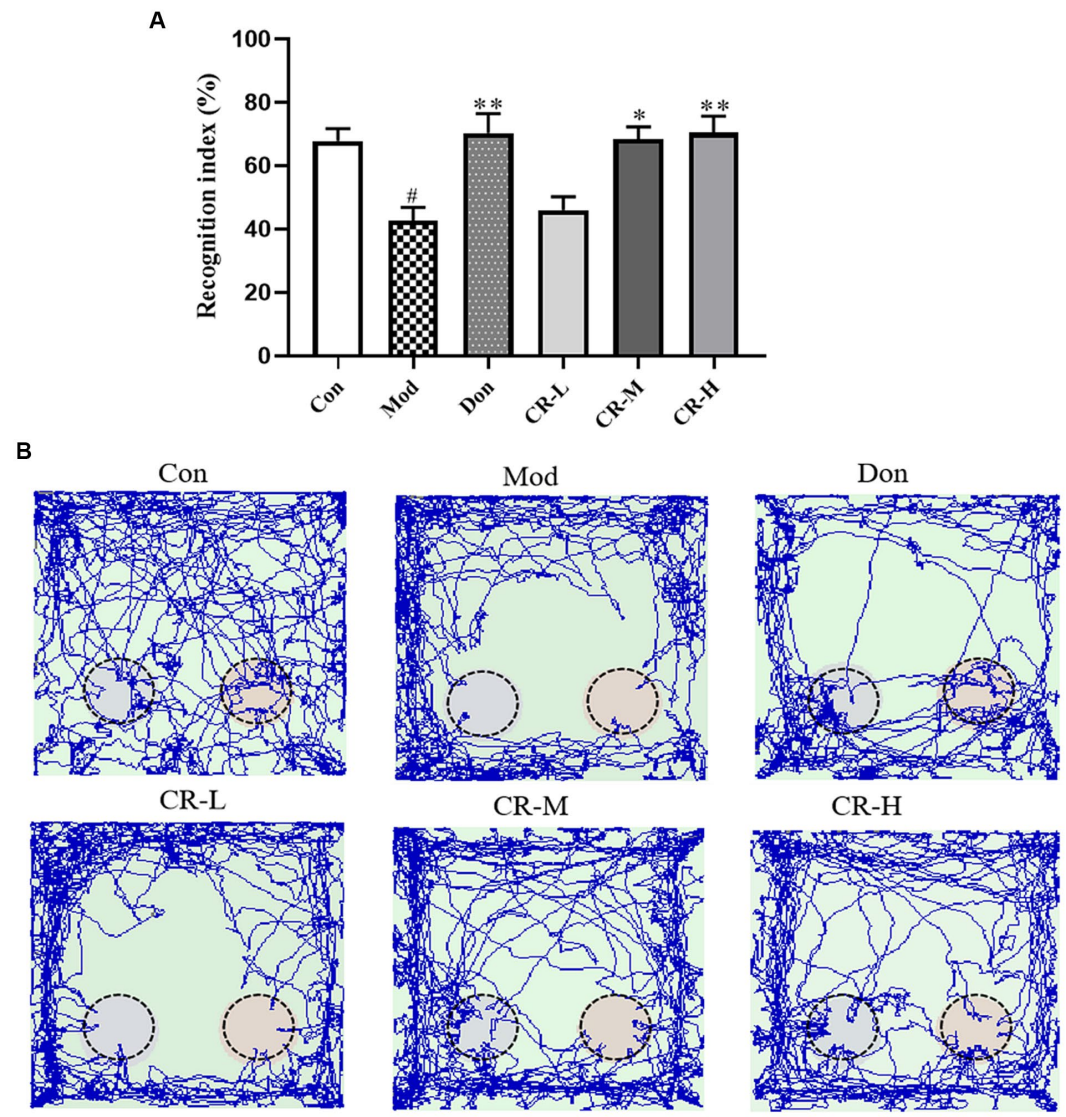
Effects of CR on the recognition index **(A)** and tracks **(B)** of mice in the NORT (Left: the new object; Right: the old object). The data were presented as mean ± SEM, *n* = 9 ~ 11; ^#^*p* < 0.05, ^##^*p* < 0.01, ^###^*p* < 0.001 vs. the Con group; ^*^p < 0.05, ^**^p < 0.01, ^***^p < 0.001, vs. the Mod group.

The results of NORT are presented in Figures 3A,B (Supplementary Table S3). Compared with the Con group, the new object recognition index of the Mod group decreased (*p* < 0.05), and AD mice had no apparent tendency to explore new objects. Compared with the AD Mod group, the new object recognition index significantly increased in the Don group (*p* < 0.01). In the CR-M and CR-H groups, the new object recognition rose in a dose-dependent manner (*p* < 0.05, *p* < 0.01). These results suggest that CR improved non-spatial recognition and memory in AD mice.

##### 3.1.1.3 Morris water maze test (MWM)

**Figure 4:**
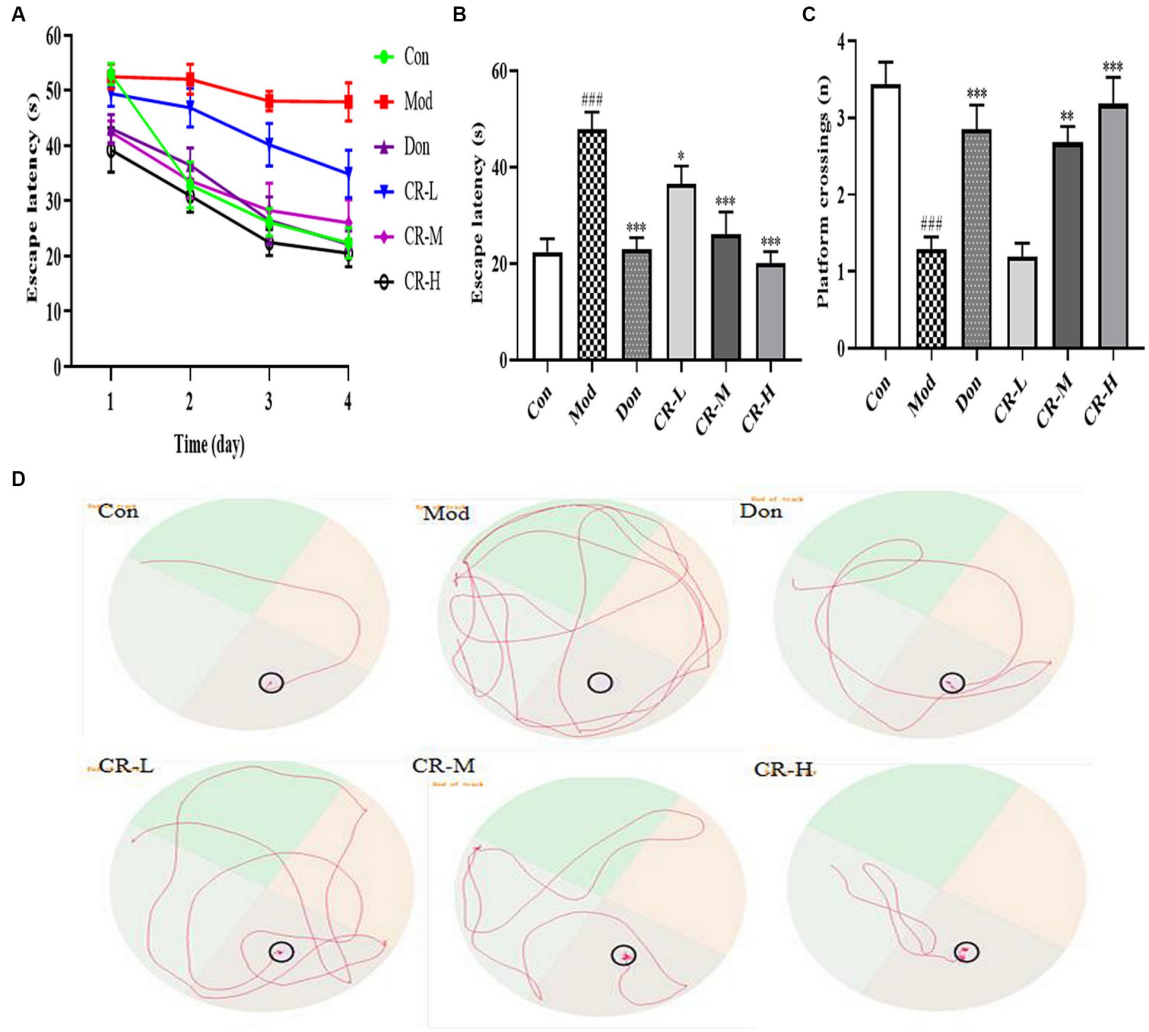
Effects of CR on the escape latency of four consecutive days test **(A)**, escape latency on the 4th day **(B)**, number of platform crossings **(C)**, and tracks on the 4th day **(D)** of mice in the MWM. The data were presented as mean ± SEM, *n* = 9 ~ 14; ^#^*p* < 0.05, ^##^*p* < 0.01, ^###^*p* < 0.001 vs. the Con group; ^*^p < 0.05, ^**^p < 0.01, ^***^p < 0.001, vs. the Mod group.

This experiment was divided into two phases, which are training and spatial probing. During the four-day training phase, the mean escape latency progressively declined in all mice, except those in the AD model group (Figure 4A). As shown in Figures 4B,D, on the 4th day of the training phase, the mice in the AD Mod group mostly adopted the search strategy of edge exploration when searching for the platform without obvious quadrant bias, compared with the Con group. Their escape latency was significantly extended to a large extent (*p* < 0.001). Compared with the Mod group, the Don, CR-L, CR-M and CR-H groups were more purposeful in searching for the platform, and their mean escape latency shortened (*p* < 0.05 or *p* < 0.001) (Figure 4B, Supplementary Table S4).

At the spatial probe phase, the number of platform crossings by AD Mod mice significantly reduced (*p* < 0.001), compared with the Con group. This indicated that D-gal & Scop impaired the learning ability, spatial memory, and scene memory acumen of the mice. Compared with the AD Mod group, the number of platform crossings in the Don, CR-M, and CR-H groups increased to different degrees (*p* < 0.01 or *p* < 0.001), especially in the Don and CR-H groups (*p* < 0.001) (Figure 4C, Supplementary Table S4). These results indicated that the CR could improve AD mice’s spatial learning memory ability.

#### 3.1.2 Nissl staining

**Figure 5:**
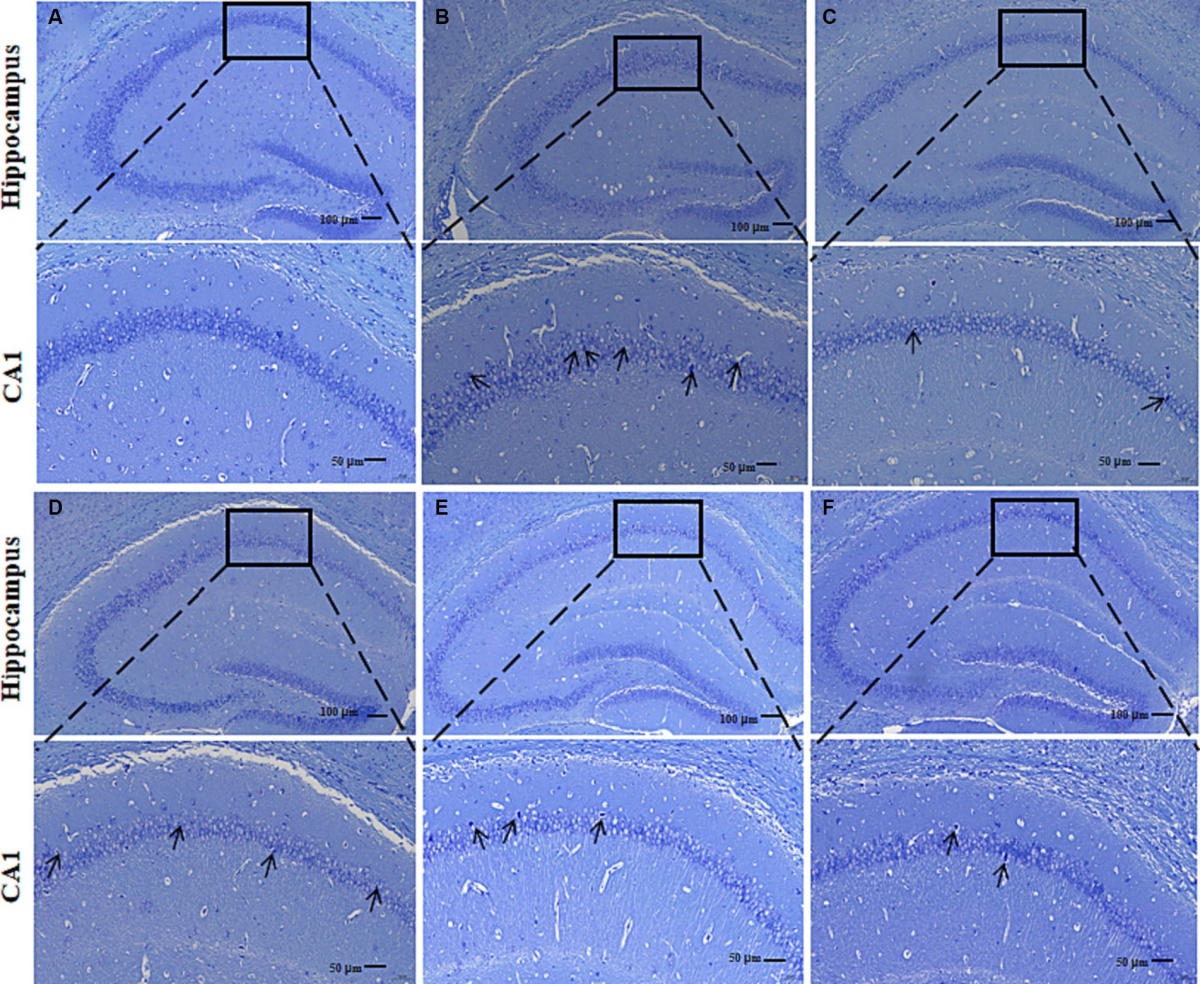
Effects of CR on the mouse hippocampal neurons. Con group **(A)**; Mod group **(B)**; Don group **(C)**; CR-L group **(D)**; CR-M group **(E)**; CR-H group **(F)**.

Nissl bodies are basophilic clusters and granules within the neuronal cytosol or dendrites, which appear blue-purple after Nissl staining. Large and abundant Nissl bodies indicate that nerve cells synthesize proteins. By staining Nissl bodies, the functional state of neurons can be intuitively reflected. In the Con group, a large number of dense pyramidal cells and granulosa cells were observed in the CA1 region of the hippocampus of mice (Figure 5A). These were neatly arranged and intact intracytoplasmic Nissl bodies. Compared with the Con group, the following observations were made in the pyramidal cells in the CA1 region of the hippocampus of the AD Mod group: autolysis of the cytoplasm; a significant reduction of Nissl bodies; vacuole-like degeneration; and deeply stained nuclei; incomplete cell morphology; loosely arranged and disordered cells, and more gaps (Figure 5B). The Nissl bodies in the CA1 region of the hippocampus of mice in Don CR-L, CR-M, and CR-H groups showed different degrees of recovery, along with increased numbers and tight arrangements (Figures 5C–F). However, in the Don and CR-H groups, the Nissl bodies assumed a full morphology and showcased the most noticeable improvement. These results indicated that CR could contribute to the recovery of Nissl bodies while reducing the degree of neuronal injury.

#### 3.1.3 Effects of CR on the levels of ACh and AChE in mice brain

**Figure 6:**
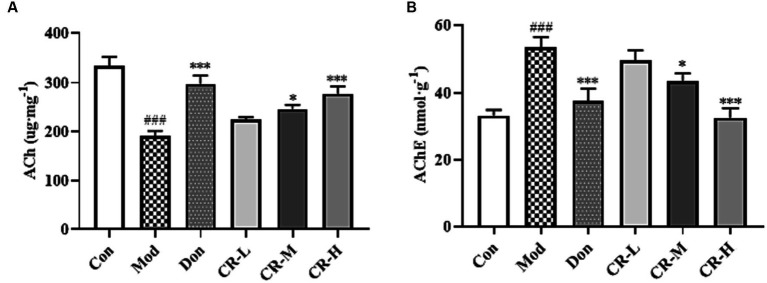
Effects of CR on the levels of ACh **(A)** and AChE **(B)** in the mice brain. The data were presented as mean ± SEM, *n* = 7; ^#^*p* < 0.05, ^##^*p* < 0.01, ^###^*p* < 0.001 vs. the Con group; ^*^p < 0.05, ^**^p < 0.01, ^***^p < 0.001, vs. the Mod group.

To assess the effects of CR on the cholinergic system, the levels of ACh and AChE in brains of the mice were measured. As shown in Figure 6 (Supplementary Table S5), the ACh level in the brains of the AD Mod group mice was significantly lower, and the AChE level was significantly higher compared with the Con group (*p* < 0.001). Compared with the AD Mod group, the Don group increased and decreased the AChE level in the mice’s brains (*p* < 0.001). There was a significant elevation in the ACh levels and reduction in AChE levels in the brains of mice in the CR-M and CR-H groups (*p* < 0.05 or *p* < 0.001). These results suggest that CR could activate the cholinergic system, improve neural signaling, and enhance cognitive functions such as learning and memory by modulating ACh and AChE levels in the brains of mice.

#### 3.1.4 Effect of CR on the levels of IL-6, IL-1β, COX-2, TNF-α, iNOS and NO in mice brain

**Figure 7:**
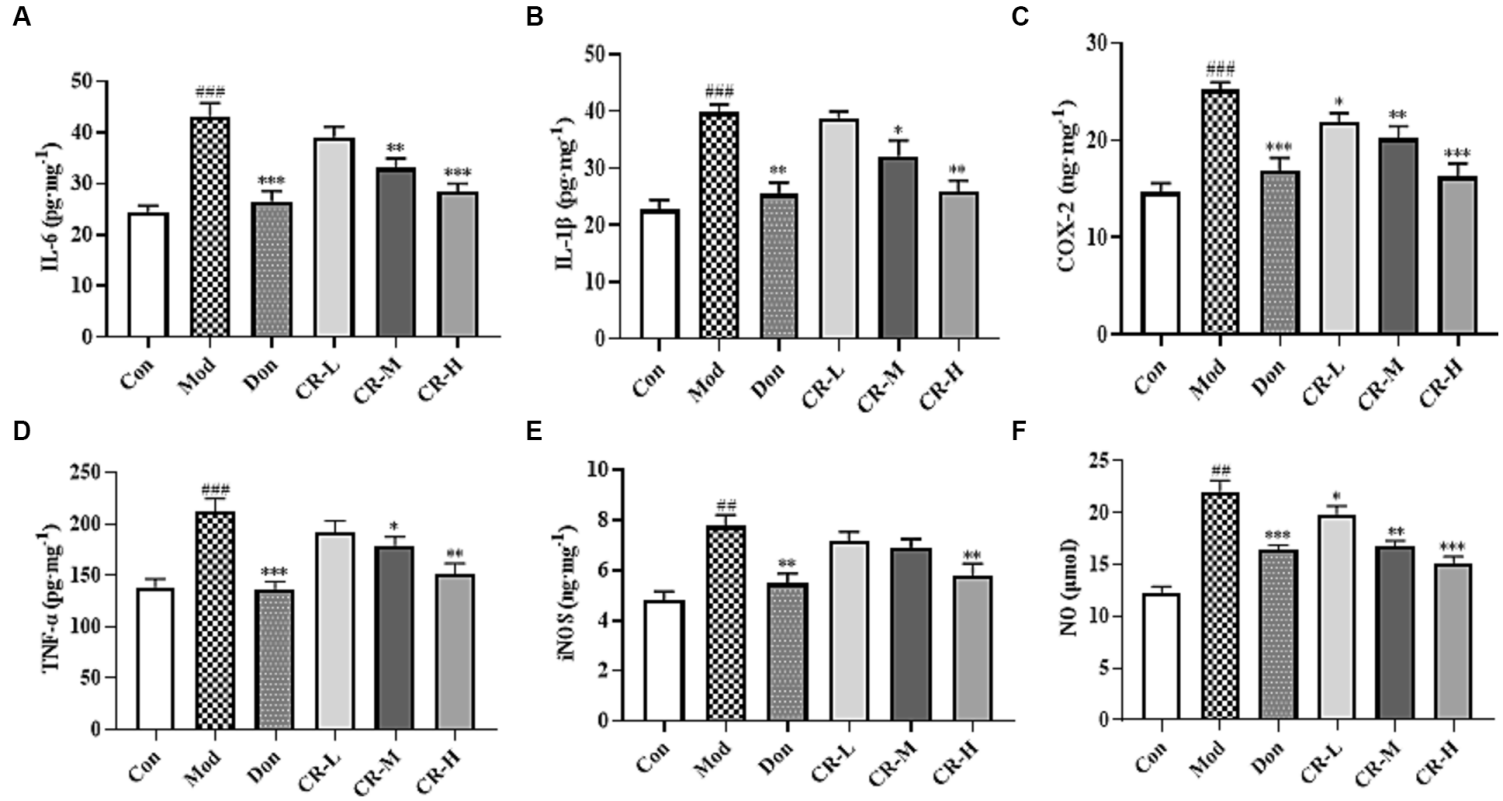
Effects of CR on the levels of IL-6 **(A)**, IL-1β **(B)**, COX-2 **(C)**, TNF-α **(D)**, iNOS **(E)**, and NO **(F)** in mice brain. The data were presented as mean ± SEM, *n* = 7; ^#^*p* < 0.05, ^##^*p* < 0.01, ^###^*p* < 0.001 vs. the Con group; ^*^p < 0.05, ^**^p < 0.01, ^***^p < 0.001, vs. the Mod group.

Figure 7 (Supplementary Table S6) shows that the levels of IL-6, IL-1β, COX-2, TNF-α, iNOS, and NO in the brains of the mice in the AD Mod group were significantly increased (*p* < 0.001 or *p* < 0.01) compared with the Con group. Comparisons with the Mod group showed that the COX-2 and NO levels in the CR-L group decreased (*p* < 0.05), the IL-6, IL-1β, COX-2, TNF-α, and NO levels in the CR-M group significantly reduced (*p* < 0.01 or *p* < 0.05), IL-6, IL-1β, COX-2, TNF-α, iNOS, and NO levels in the Don and CR-H groups also significantly decreased (*p* < 0.001 or *p* < 0.01). These findings indicate that CR could reduce the neuroinflammatory response that is induced by D-gal & Scop.

#### 3.1.5 Effect of CR on the levels of MDA, SOD, and GSH-Px in mice brain

**Figure 8:**
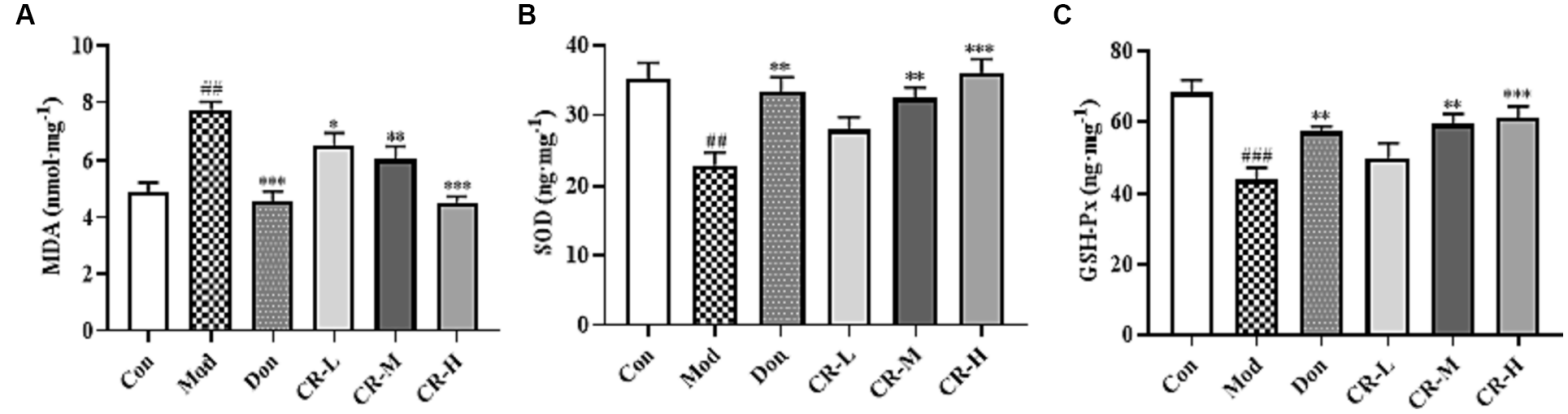
Effects of CR on the levels of MDA **(A)**, SOD **(B)**, and GSH-Px **(C)** in mice brain. The data were presented as mean ± SEM, *n* = 7; ^#^*p* < 0.05, ^##^*p* < 0.01, ^###^*p* < 0.001 vs. the Con group; ^*^p < 0.05, ^**^p < 0.01, ^***^p < 0.001, vs. the Mod group.

To evaluate the effects of CR on the oxidative stress induced by D-gal & Scop, the MDA, SOD, and GSH-Px levels in mice brains were measured. The results are presented in Figure 8 (Supplementary Table S7). Compared with the Con group, the MDA levels in the Mod group of mice were significantly higher (*p* < 0.01), while those of SOD and GSH-Px showed a significant decrease (*p* < 0.01 or *p* < 0.001). Based on comparisons with the Mod group, the following findings were observed: the MAD levels were reduced in the CR-L group (*p* < 0.05); CR-M significantly decreased the MDA level and elevated the SOD and GSH-Px levels (*p* < 0.01); and the CR-H group had significant reductions in MDA levels, accompanied by increases in SOD and GSH-Px levels (*p* < 0.001).

### 3.2 Identification of absorbed alkaloids

**Figure 9:**
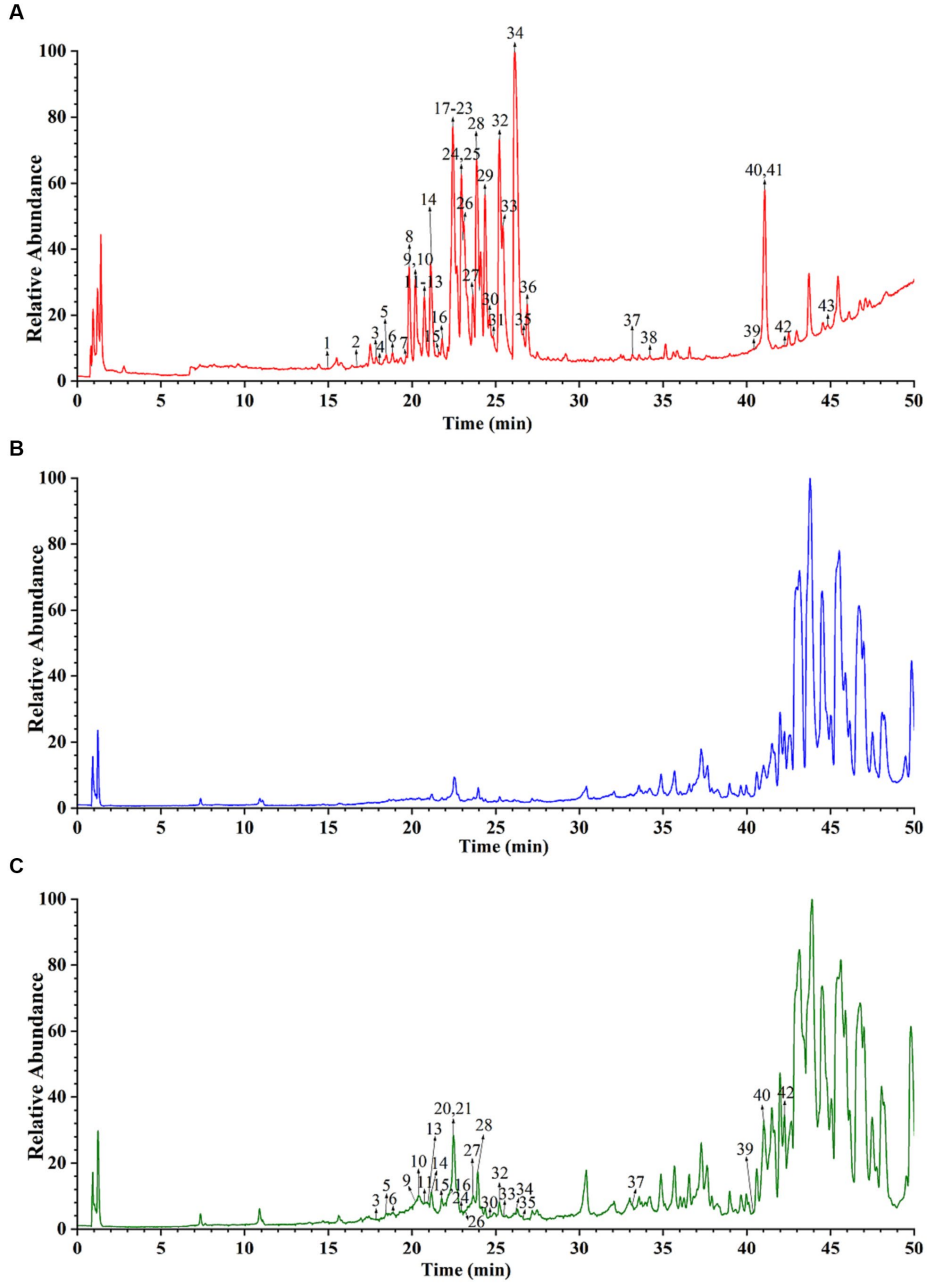
Total ion chromatograms of CR sample **(A)**, blank serum **(B)**, and drug-containing serum **(C)**.

The main active components in CR are alkaloids. Thus, this study used the positive ion scanning mode to obtain each sample’s total ion chromatograms. To identify the active alkaloid component of CR, a deeper understanding of the alkaloid composition that enters the serum after oral administration of CR is required. Comparisons of the total ion chromatography of CR, blank serum, and drug-containing serum under the same UPLC and MS conditions were done to distinguish the prototypical components of CR in the serum (Figure 9). A total of 25 alkaloid prototype constituents were identified, including D-isoboldine, corypalmine, thaliporphine, yuanhunine, tetrahydrocolumbamine, dl-Tetrahydrocoptisine, protopine, tetrahydropalmatine, coptisine, (R)-canadine, and palmatine. Among the 25, 15 were protoberberine alkaloids, and the rest were aporphine and protopine alkaloids. This suggests that protoberberine alkaloids might be one of the key alkaloid types of CR involved in treating AD. The detailed information is available in Supplementary Table S8.

### 3.3 Network pharmacology

A total of 18 candidate bioactive components were screened based on the TCMSP database, combined with serum pharmacochemistry. The detailed information is available in Supplementary Table S9.

**Figure 10:**
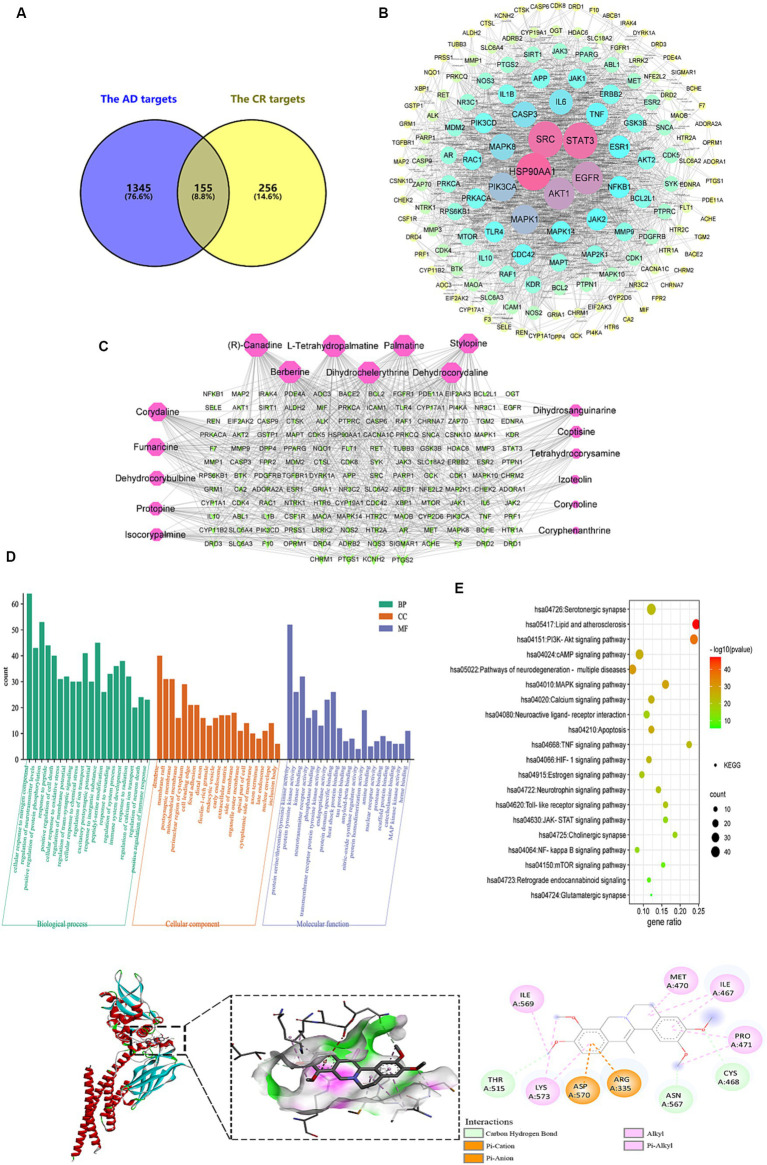
Analysis of targets potentially affected by CR treatment. The intersection of AD and CR targets **(A)**; PPI network of 155 intersecting targets, the color and size of the node correlate with its degree value **(B)**; the candidate bioactive component-target network, red nodes represent the candidate bioactive component of CR, green nodes represent intersecting targets, the size of node correlates with its degree value **(C)**; GO **(D)** and KEGG pathway **(E)** enrichment analysis results of the intersecting targets; the binding pattern of STAT3 with dehydrocorydaline **(F)**.

A sum of 411 non-duplicated targets of the candidate bioactive components was obtained from TCMSP, ETCM, and Swiss Target Prediction databases. A total of 1,500 AD-related targets were collected from the GeneCards, TTD, and OMIM databases. There were 155 AD-related common targets that were identified in the Venn diagram (Figure 10A). These were putative targets of CR against AD.

The PPI network with 155 common targets was constructed (Figure 10B). There were 148 nodes (hiding seven unconnected nodes) and 858 edges on the PPI network. The average connectivity and local clustering coefficient of the nodes were 11 and 0.482, respectively. The results suggested that there was a strong correlation between each target. In this network, the darker the color, the larger the node, indicating that the higher the target degree, the more critical it is. HSP90AA1, SRC, STAT3, EGFR, and AKT1 might be CR’s core targets for treating AD (Supplementary Table S10).

The topological property analysis of the PPI network was performed using the Centiscape 2.2 plugin of Cytoscape. There were 33 key targets with Closeness >0.0158, Betweenness >261.53, and Degree >22.88, accounting for 22.3% of all targets. Half of the key targets were related to inflammation and immunity. Therefore, regulating the inflammatory response is a critical mechanism through which CR may treat AD.

The candidate bioactive component-target network contained 166 nodes and 591 edges, and the average connectivity was 7.12 (Figure 10C). From the figure, it is clear that the complex network relationship of “one component-multiple target” and “one target-multiple component” indicates the multi-component and multi-target synergistic effects of CR against AD. The top-10 component nodes that were linked to more target nodes were (R)-canadine, L-tetrahydropalmatine, palmatine, stylopine, berberine, dihydrochelerythrine, dehydrocorydaline, corydaline, fumaricine and dehydrocorybulbine, which might be the core components of CR involved in the treatment of AD.

With *p* < 0.01 as the condition for screening pathways, the common targets between candidate bioactive components and AD were further analyzed for GO function and KEGG signal pathway enrichment analysis using Metascape. The top-20 pathways were extracted for mapping, and the results are shown in Figures 10D,E and Supplementary Table S11.

CR anti-AD was mainly involved in biological processes such as cellular response to nitrogen compound, regulation of neurotransmitter levels, protein phosphorylation, response to peptide, positive regulation of cell death, cellular response to oxidative stress, regulation of membrane potential, and trans-synaptic signaling (Figure 10D). Cellular components were primarily found in dendrites, membrane rafts, postsynaptic membranes, and mitochondrial membranes. Molecular functions were mainly revealed in protein kinase activity, neurotransmitter receptor activity, phosphoprotein binding, heat shock protein binding, tau and amyloid-beta protein binding, and nitric-oxide synthase regulator activity.

The KEGG pathway enrichment analysis is shown in Figure 10E. The pathways that were associated with oxidative stress were the HIF-1 signaling and mTOR signaling pathways. The JAK–STAT signaling, NF-kappa B signaling, TNF signaling, and Toll-like receptor signaling pathways were related to neuroinflammation. The pathways that were associated with neurotransmitters included neuroactive ligand-receptor interaction, neurotrophin signaling pathway, cholinergic synapse, glutamatergic synapse, and serotonergic synapse. Those that were related to apoptosis were the PI3K-Akt and MAPK signaling pathways. Signaling-related pathways included calcium, cAMP, and retrograde endocannabinoid signaling. The estrogen signaling pathway was associated with hormonal regulation. It was suggested that the bioactive components of CR could regulate these pathways through multiple targets, thereby enhancing anti-oxidative stress attributes, anti-neuroinflammation, regulation of neurotransmitter levels and apoptosis, in addition to other effects that promote the synergistic treatment of AD. Further analysis of the KEGG results revealed that most of the selected pathways were related to inflammatory responses. In fact, pathways such as JAK–STAT, NF-κB, TNF, and Toll-like receptor (TLR4) interacted to influence the expression of their downstream IL-6, IL-1β, TNF-α, COX2, and other inflammatory factors.

### 3.4 Molecular docking

After obtaining the core active components and key targets of CR, Auto Dock Vina 1.1.2 was used to perform receptor-ligand docking simulation calculations between the core active components and STAT3, which is closely related to the inflammatory pathway in the core targets. Generally, if the binding energy is less than −1.2 kaL·moL^−1^, then the small molecules can freely bind to proteins. When the binding energy is less than −5.0 kaL·moL^−1^, the interaction between small molecules and proteins is stronger. The binding energy of less than −7.0 kaL·moL^−1^ indicates that the small molecule interacts strongly with the protein. Therefore, the lower the binding energy, the stronger the interaction.

The molecular docking results of 10 core active components of CR (tetrahydroberberine, tetrahydropalmatine, Palmatine, Tetrahydrocoptisine, Berberine, Dihydrochelerythrine, Dehydrocorydalin, Corydaline, Fumaricine, and Dehydrocorybulbine) to the STAT3 target protein are shown in Table 1. All binding energies were less than −5.0 kaL·moL^−1^, indicating that these components had a strong binding ability with the STAT3 target protein. Dehydrocorydaline exhibited the strongest binding capacity to the STAT3 target protein and might be the most potential key compound for the prevention and treatment of AD. As an example, the binding pattern of dehydrocorydaline is shown in Figure 10F. Dehydrocorydaline can bind to the pocket of STAT3 protein, where THR515, ASN567, and CYS468 amino acid residues interact with it via carbon hydrogen bonds. The ASP570 and ARG335 amino acid residues interact with it in modes such as Pi-Cation or Pi-Anion.

**Table 1:** Molecular docking results for core active components with the STAT3 target protein.

MOL ID	Compound name	Docking energy (kal·moL^−1^)
MOL002903	(R)-Canadine	−7.0
MOL004071	L-Tetrahydropalmatine	−7.0
MOL000785	Palmatine	−7.0
MOL004230	Stylopine	−7.8
MOL001454	Berberine	−7.1
MOL001461	Dihydrochelerythrine	−7.3
MOL004204	Dehydrocorydaline	−8.0
MOL004195	Corydaline	−6.6
MOL004210	fumaricine	−7.8
MOL004203	Dehydrocorybulbine	−7.4

### 3.5 Results of *in vitro* cellular validation

#### 3.5.1 CR may inhibit the production of pro-inflammatory mediators and promote the production of anti-inflammatory mediator in BV2 cells

**Figure 11:**
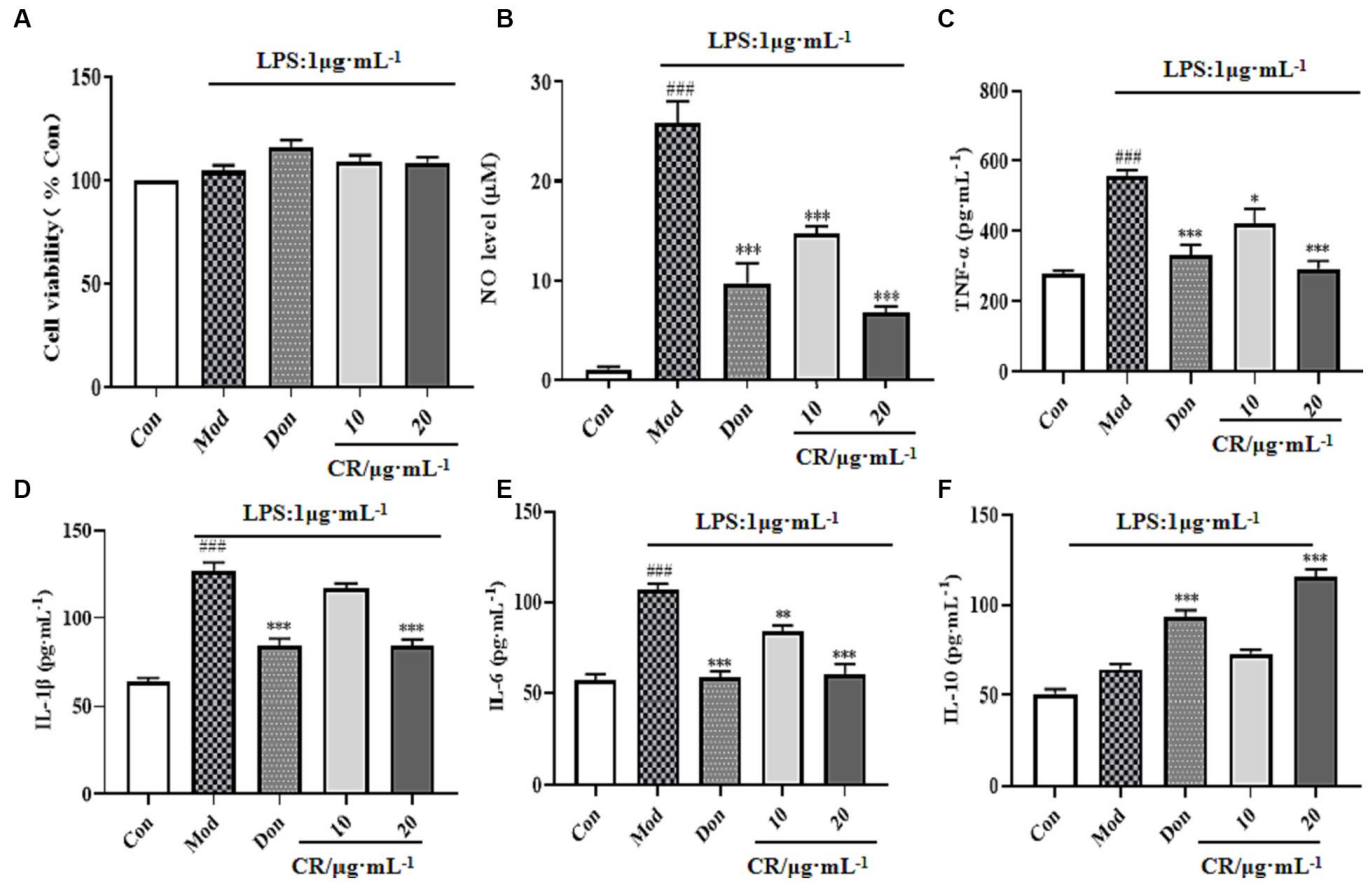
Effects of CR on the levels of inflammatory mediators in the supernatant of LPS-induced BV2 cells (Supplementary Table S12). Cell viability **(A)**, NO level **(B)**, TNF-α level **(C)**, IL-1β level **(D)**, IL-6 level **(E)**, IL-10 level **(F)**. The data were presented as mean ± SEM, *n* = 5; ^#^*p* < 0.05, ^##^*p* < 0.01, ^###^*p* < 0.001 vs. the Con group; ^*^p < 0.05, ^**^p < 0.01, ^***^p < 0.001, vs. the Mod group.

Compared to the Con group, LPS, Don, and different concentrations of CR did not affect the viability of BV2 cells in each group at experimental concentrations (Figure 11A). Inflammatory mediators play critical roles in numerous neuronal disorders. To examine the effects of CR on LPS-induced BV2 cells, the production of NO, TNF-α, IL-1β, IL-6, and IL-10 in the supernatant of cells from the Mod group was measured. The findings showed that LPS induced an obvious NO production from the BV2 cells, which was significantly reversed by treatment with CR (10, 20 μmol·L^−1^) compared with the Mod group (Figure 11B) (*p* < 0.001). The extent of the reversal was dose-dependent. As a positive control, Don similarly attenuated NO production at a concentration of 40 μmol·L^−1^. It was also observed that LPS treatment increased TNF-α, IL-1β, and IL-6 the levels compared with the Con group (*p* < 0.001), whereas Don and CR (10, 20 μmol·L^−1^) effectively prevented the production of TNF-α and IL-6 in a dose-dependent manner (*p* < 0.05 or *p* < 0.01) or (*p* < 0.001) (Figures 11C,E). Don and CR (20 μmol·L^−1^) significantly lowered IL-6 levels (*p* < 0.001) (Figure 11D) and increased IL-10 levels (*p* < 0.001) (Figure 11F). These results suggest that CR may inhibit the production of pro-inflammatory mediators and promote the production of anti-inflammatory mediators.

#### 3.5.2 CR exerts anti-neuroinflammatory effects by inhibiting the JAK2-STAT3 pathway in LPS-induced BV2 cells

**Figure 12:**
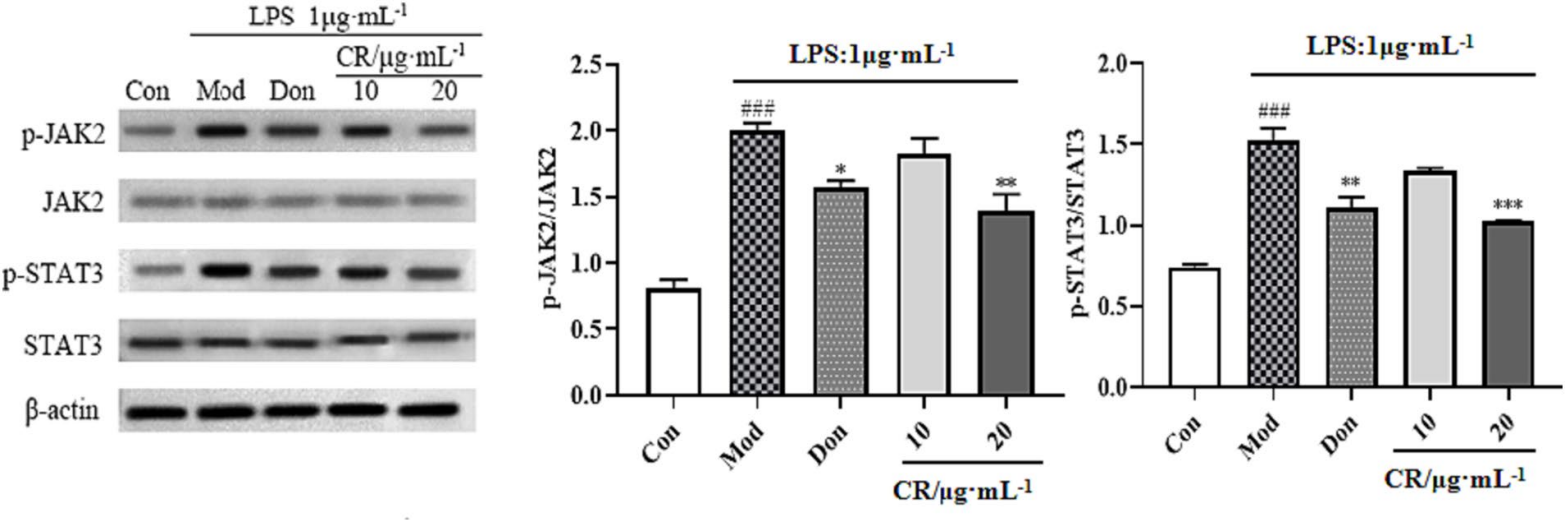
Effects of CR on p-JAK2/JAK2 and p-STAT3/STAT3 ratios in BV2 cells. The data were presented as mean ± SEM, *n* = 3; ^#^*p* < 0.05, ^##^*p* < 0.01, ^###^*p* < 0.001 vs. the Con group; ^*^p < 0.05, ^**^p < 0.01, ^***^p < 0.001, vs. the Mod group.

The JAK2/STAT3 signaling pathway is vital for activating microglia and inflammatory mediator production. Therefore, the regulatory effects of CR on activating the JAK2/STAT3 pathway were investigated. As shown in Figure 12 (Supplementary Table S13), the phosphorylation levels were upregulated in response to the LPS stimulus compared with the Con group (*p* < 0.001). Moreover, activating JAK2 subsequently triggers STAT3 transcription factor phosphorylation and dimerization. In the present study, LPS markedly elevated the phosphorylated levels of STAT3 while p-JAK2/JAK2 and p-STAT3/STAT3 ratios increased (*p* < 0.001). These results implied that JAK2/STAT3 pathway was activated in the LPS-induced BV2 cells. Compared with the Mod group, Don and CR (20 μmol·L^−1^) significantly down-regulated the phosphorylation levels of JAK2 and its downstream signaling mediator STAT3. Both p-JAK2/JAK2 and p-STAT3/STAT3 ratios were reduced (*p* < 0.05 or *p* < 0.01 or *p* < 0.001), indicating that CR effectively blocked the activation of the JAK2/STAT3 pathway. From this information, we speculated that the inhibitory effects of CR on LPS-induced neuroinflammation are through, at least in part, the JAK2/STAT3 signaling pathway.

#### 3.5.3 CR can suppress phenotypic M1 polarization and promote phenotypic M2 polarization in LPS-activated BV2 cells

**Figure 13:**
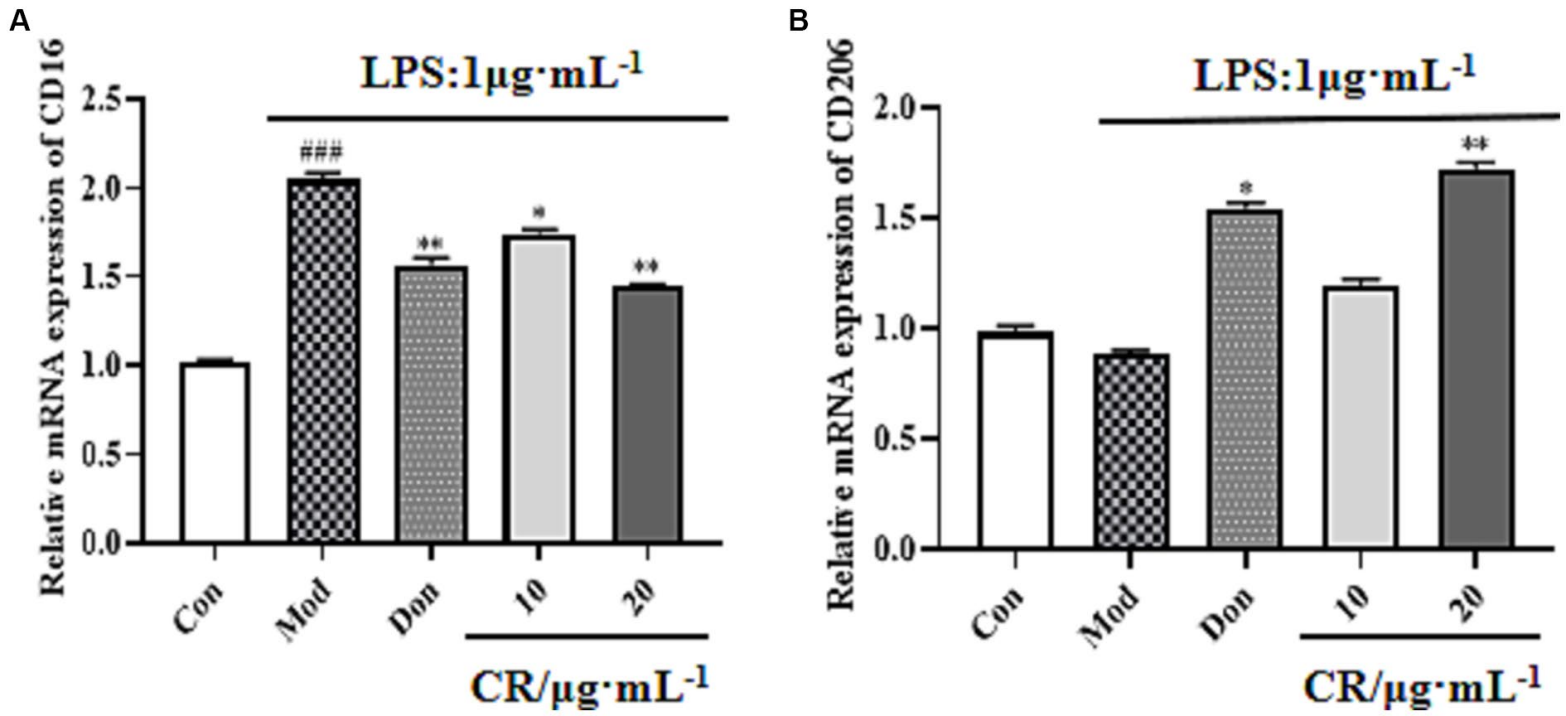
Effects of CR on M1 marker (CD16) and M2 marker (CD206) mRNA expression in BV2 cells. The relative mRNA expression of CD16 **(A)**. The relative mRNA expression of CD206 **(B)**. The data were presented as mean ± SEM, *n* = 3; ^#^*p* < 0.05, ^##^*p* < 0.01, ^###^*p* < 0.001 vs. the Con group; ^*^p < 0.05, ^**^p < 0.01, ^***^p < 0.001, vs. the Mod group.

LPS is regarded as a classical M1 microglia inducer, which causes phenotypic M1 expression of pro-inflammatory cytokines. Findings from this study showed that the mRNA expression of CD16 (M1 marker) increased in LPS-activated BV2 cells compared with the Con group (*p* < 0.001). Compared with the Mod group, CR could inhibit the mRNA expression of M1 markers under inflammatory stimulus conditions (Figure 13A, Supplementary Table S14) and promote the transition of activated microglia to phenotype M2, as evidenced by an increase in the expression of CD206 (Figure 13B, Supplementary Table S14).

## 4 Discussion

The pathogenesis of AD is controversial, and single-target drugs may fail to treat this multifactorial disease. However, modulation of various paths of action through multi-targeting may aid better outcomes for AD treatment. Indeed, multi-target strategies have been practiced in clinics and clinical trials (Cheong et al., 2022). Based on their complex composition, Chinese medicines are potent interventions against diseases, owing to the multiple target or pathway interactions that they enhance. This attribute of Chinese medicines is a treasure trove for developing multi-targeted anti-AD drugs.

### 4.1 Study of CR efficacy in treating AD

We used the AD mouse model induced by D-gal & Scop to investigate the pharmacodynamic effects of CR anti-AD based on behavioral changes, brain histopathological sections, the cholinergic system, oxidative stress, and neuroinflammatory responses. Donepezil hydrochloride (Don), is one of the main drugs for the clinical therapy of AD. It is a long-acting cholinesterase inhibitor, which can enhance the learning, memory, and other cognitive functions of the mild or moderate AD patients, reducing the degree of dementia (Xia et al., 2023). Relevant studies have shown that Don also has pharmacologic effects such as anti-neuroinflammation and attenuation of oxidative stress (Guo et al., 2015; Kim et al., 2021; Elbaz et al., 2023). Hence, the Don was chosen as a positive drug for comparing the efficacy of CR as an anti-AD intervention and neuroinflammation inhibitor *in vitro*.

The OFT, NOR, and MWM results showed that the CR improved AD mice’s anxiety-like behavior, spatial and non-spatial recognition, and memory acumen. The brain’s hippocampus region is related to learning, memory, and cognitive functions. The CA1 region is considered a key site for forming long-term potentiation effects. The CA1 region of AD patients often exhibits significant atrophy and has fewer neurons and synapses (Bird and Burgess, 2008). The results of Nissl staining suggested that CR contributed to the recovery of Nissl bodies in the CA1 region while also reducing the degree of neuronal injury.

The cholinergic system is one of the most critical neurotransmitter systems in learning and memory. ACh is the primary cholinergic neurotransmitter, and AChE is a key enzyme responsible for hydrolyzing ACh. CR reduced the AChE levels and increased the Ach concentrations in the brains of AD mice. This activated the cholinergic system and enhanced cognitive functions such as learning and memory.

IL-6, IL-1β, and TNF-α are classical proinflammatory cytokines. COX-2 is one of the important markers of inflammation and neurological lesions (Rauf et al., 2022). The high expression of iNOS causes overproduction of the neurotransmitter NO. Elevated levels of both imply worsening nerve cell damage and neuroinflammation (Yuan et al., 2015). This study showed that the concentrations of IL-6, IL-1β, COX-2, TNF-α, iNOS, and NO in the brain of AD mice significantly increased, and that the intervention of CR could reverse the abnormal changes in inflammatory markers. This suggests that CR could treat AD by inhibiting neuroinflammation.

The *in vivo* MDA level is one of the critical indicators for detecting oxidative stress and damage. SOD and GSH-Px are essential components of the antioxidant defense system in the brain tissue. Reducing oxidative stress has been an important strategy for treating AD (Cioffi et al., 2021; Bai et al., 2022). This study showed that D-gal & scop could have the following effects in the brain of mice: induce oxidative stress in mice; significantly increase the levels of MDA, which is the product of oxidative damage; and decrease the content of SOD and GSH-Px, which are the antioxidant markers. CR could reverse the imbalance of oxidative stress markers and reduce the related stress and damage, thereby playing a neuroprotective role.

### 4.2 Analysis of the core active components in CR

We speculated that tetrahydroberberine, tetrahydropalmatine, palmatine, tetrahydrocoptisine, berberine, dihydrochelerythrine, dehydrocorydalin, corydaline, fumaricine, and dehydrocorybulbine might be the core active components of CR in treating AD.

All these components, except dihydrochelerythrine and fumaricine (less studied for their pharmacological activity), belong to the protoberberine type of alkaloids, which are a kind of isoquinoline alkaloids. Many preclinical studies revealed that these alkaloids were helpful and had great anti-AD potential (Meng et al., 2018; Vrabec et al., 2023). For example, Kilic et al. (2021) found that the protoberberine type of alkaloids was the most effective inhibitor of AChE and Buche, after comparing the anti-cholinesterase activity of different alkaloids *in vitro*. Palmatine, berberine, and dehydrocorydaline have excellent inhibitory effects on AChE (Xiao et al., 2011). Inhibiting AChE activity and affecting neurotransmitter release is still the primary therapeutic strategy for AD. Additionally, berberine, tetrahydroberberine, tetrahydrocoptisine, tetrahydropalmatine, and palmatine can prevent and treat AD by inhibiting inflammation, oxidative stress, and Aβ aggregation (Kulkarni and Dhir, 2010; Qu et al., 2016). Glutamate is an excitatory neurotransmitter involved in various neural functions such as learning, memory, and synaptic plasticity. Lin et al. (2022) found that dehydrocorydaline could protect neurons from damage by inhibiting the MAPK/ERK/synapsin signaling pathway, reducing the increase of Ca^2+^, and regulating glutamate release. Although there are no studies that demonstrate the anti-AD effects of dihydrochelerythrine, fumaricine, and dehydrocorybulbine, we speculated that they might possess the potential. More attention could be given to the above compounds and the protoberberine-type alkaloids in the future efforts to discover, design, and synthesize new anti-AD drugs.

### 4.3 Analysis of key targets and mechanisms for CR in treating AD

Based on analyzing the PPI network of CR anti-AD active targets, HSP90AA1, Src, and STAT3 were the top three targets. The Heat shock protein 90α (Hsp90α), which is encoded by the HSP90AA1 gene, is primarily responsible for stress-induced functions such as auxiliary protein folding, complex assembly, and degradation, which are critical for the homeostasis of client proteins (Zuehlke et al., 2015). Extracellular Hsp90α can induce inflammatory responses by activating STAT3 and NF-κB transcriptional programs (Bohonowych et al., 2014). HSP90AA1 is identified as a marker of microglia activation in idiopathic Parkinson’s disease (Smajic et al., 2022). The Src protein is a non-receptor protein tyrosine kinase family. It’s a typical signaling enzyme in neuronal growth cones (Dhawan and Combs, 2012). When neurons are damaged, their expression is up-regulated, while proinflammatory cytokine transcription and free radical production are activated. This exacerbates neurodegeneration. Meanwhile, Src can also induce the phosphorylation of tyrosine residues, in addition to activating STAT3.

Both Hsp90α and Src can activate downstream STAT3 (Hu et al., 2021). STAT3 is predominantly expressed in brain tissue, with high levels being observed in the autopsy brain tissue of AD patients (Reichenbach et al., 2019; Rusek et al., 2023). Another study showed that STAT3 is involved in Aβ-induced chronic activation of microglia and the release of inflammatory factors. In Aβ-induced AD transgenic mice, STAT3 expression increased, suggesting that STAT3 may be closely associated with chronic inflammatory damage in AD patients (Xiong et al., 2014). Subsequently, molecular docking of the core active components of CR with STAT3 was undertaken, and all the binding energies were below −5.0 kaL moL^−1^. This indicated that the components strongly interacted with STAT3. Dysregulation of the JAK/STAT signaling pathway can lead to inflammation, immunodeficiency syndromes, cancer, and neurodegenerative diseases (O’Shea et al., 2015). Given this, we infer that the JAK2/STAT3 pathway plays an essential role in the treatment of AD with CR. This is consistent with the results from KEGG enrichment analysis. The path was finally validated using the LPS-induced BV2 inflammation model.

### 4.4 Validation of the anti-inflammatory mechanism of CR

Elevated levels of inflammatory markers in AD patients and the discovery of AD risk genes associated with innate immune function point to the fact that neuroinflammation is critical in the progression of AD (Chen and Holtzman, 2022). Furthermore, neuroinflammation can vastly contribute to the proliferation of crucial pathological proteins in the brain. It is a critical link integral to the upstream pathogenesis of AD (Pascoal et al., 2021). Although treatment with NSAIDs reduces the risk of AD (Vlad et al., 2008), intervening in AD progression by targeting neuroinflammation may be an effective strategy.

Microglia are resident immune cells in the central nervous system (CNS) and they are a primary source of brain inflammation (Wang C. et al., 2023). Microglia can be divided into the resting (M0) and activation (pro-inflammatory phenotype M1 and anti-inflammatory phenotype M2) phases. M0 monitors changes in the brain parenchymal microenvironment. Activated microglia are in a dynamic equilibrium between M1 and M2 and play a “double-edged sword” role in AD pathogenesis. M1 (phenotypic marker CD16) releases pro-inflammatory factors such as TNF-α, IL-6, and IL-1β, which can damage neighboring neurons (Colonna and Butovsky, 2017). Contrastingly, M2 (phenotypic marker CD206) secretes anti-inflammatory and neurotrophic factors, including IL-10 and TGF-β, which have trophic and regenerative effects on neurons (Hu et al., 2015). IL-6 is considered one of the main coordinators of the inflammatory response in the brain, and its signaling is coordinated by STAT3 through a JAK2-dependent mechanism (Yang et al., 2022). As a downstream effector of the JAK2/STAT3 pathway, IL-6 can activate this pathway and induce microglia to polarize to M1 (Zhong et al., 2022).

**Figure 14:**
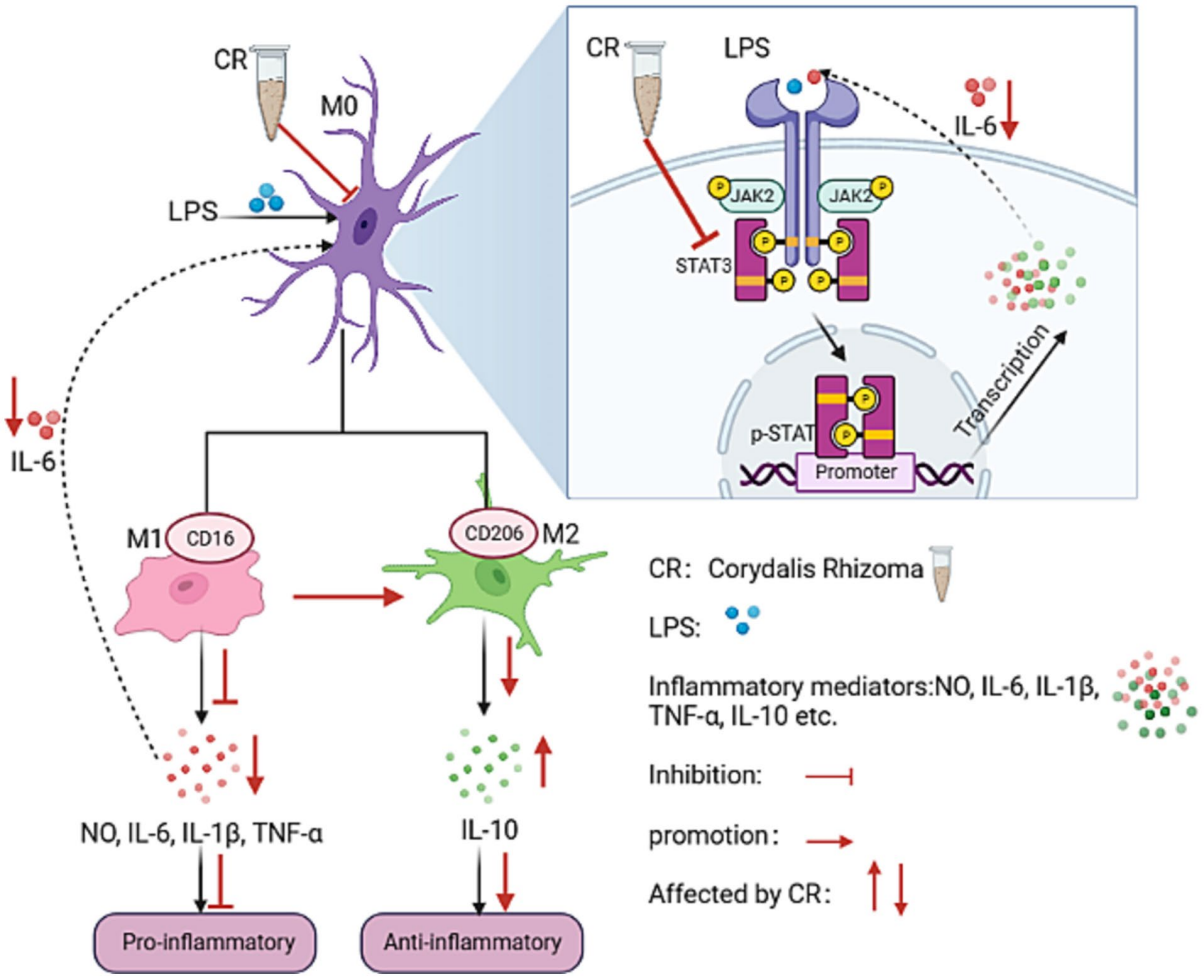
The potential anti-neuroinflammatory mechanism of CR on BV2 microglia.

The BV2 cell line is a mouse microglial cell line that replicates the situation *in vivo*, with high fidelity under inflammatory conditions (Henn et al., 2009). This cell line has been widely used in studying neuroinflammatory mechanisms in AD progression. In this study, the levels of IL6, TNF-α, IL-1β, p-JAK2, and p-STAT3 were significantly elevated in LPS-induced BV2 cells. These findings are consistent with reports that are available in literature (Li et al., 2016). After CR intervention, the levels of M1 microglia-associated markers (IL-6, TNF-α, and IL-1β) and CD16 mRNA expression significantly reduced. The concentrations of M2 microglia-associated markers, IL-10, and CD206 mRNA expression significantly increased. A significant down-regulation of the phosphorylation levels of p-JAK2 and p-STAT3 was also observed. For the first time, the present study demonstrated that CR alleviates neuroinflammation by inducing the polarization of LPS-activated BV2 microglia from phenotype M1 to phenotype M2. This partly happens through modulation of the IL-6/JAK2/STAT3 signaling pathway. The mechanism of action is shown in Figure 14.

## 5 Conclusion

The findings from this study proved that CR enhanced the co-treatment of AD by regulating neurotransmitter levels, anti-neuroinflammation, and anti-oxidative stress. Serum pharmacology combined with network pharmacology revealed that (R)-canadine, L-tetrahydropalmatine, palmatine, stylopine, berberine, dihydrochelerythrine, dehydrocorydaline, corydaline, fumaricine, and dehydrocorybulbine may be the core alkaloidal components of CR involved in treating AD. Finally, one of the important pathways predicted by network pharmacology was validated by the LPS-induced inflammation model in BV2 cells. The results showed that CR modulated the polarization of BV2 cells from the pro-inflammatory phenotype M1 to the anti-inflammatory phenotype M2 type. This is partly made possible by regulating the IL-6/JAK2/STAT3 pathway, thereby alleviating inflammation and ultimately inhibiting the progression of AD. This study not only proved that CR’s therapeutic effects against AD and its potential application in secondary development, it also provided a reference for the development of alkaloidal drugs for the treatment of AD. This study also offered the data that supports CR intervention in the progression of AD, through its anti-inflammation attributes.

## Data availability statement

The datasets presented in this study can be found in online repositories. The names of the repository/repositories and accession number(s) can be found in the article/Supplementary material.

## Ethics statement

The animal studies were approved by Animal Care and Use Committee of Beijing University of Chinese Medicine. The studies were conducted in accordance with the local legislation and institutional requirements. Written informed consent was obtained from the owners for the participation of their animals in this study.

## Author contributions

YL: Data curation, Writing – original draft, Conceptualization. YW: Writing – original draft. JG: Writing – review & editing. YqW: Formal analysis, Writing – review & editing. YLu: Validation, Writing – review & editing. ZH: Methodology, Visualization, Writing – review & editing. TJ: Data curation, Validation, Writing – review & editing. WF: Investigation, Writing – review & editing. YLi: Software, Writing – review & editing. JS: Conceptualization, Funding acquisition, Project administration, Supervision, Writing – review & editing.

## Footnotes

^1^ https://tcmspw.com/tcmsp.php

^2^ http://www.swisstargetprediction.ch/

^3^ http://www.tcmip.cn/ETCM/index.php/

^4^ https://www.genecards.org

^5^ http://bidd.nus.edu.sg/group/cjttd

^6^ http://www.omim.org

^7^ https://string-db.org/

^8^ http://metascape.org/gp/index.html

^9^ http://www.rcsb.org/pdb/home.do
