# Supplementary material for: Integrating serum pharmacochemistry and network pharmacology to reveal the active constituents and mechanism of Corydalis Rhizoma in treating Alzheimer’s disease
Source: Front Aging Neurosci. 2023 Nov 22;15:1285549. doi: 10.3389/fnagi.2023.1285549 (PMC10702756; doi:10.3389/fnagi.2023.1285549)
Supplement: Supplementary file 1 [file Data_Sheet_1.docx]

Supplementary Material

# Supplementary Tables

**Table S1.** Primer sequences for RT-qPCR

| Primer | Forward primer sequence | Reverse primer sequence |
| --- | --- | --- |
| CD16 | F: AATGCACACTCTGGAAGCCAA | R: CACTCTGCCTGTCTGCAAAAG |
| CD206 | F: TTCAGCTATTGGACGCGAGG | R: GAATCTGACACCCAGCGGAA |
| GAPDH | F: CCTCGTCCCGTAGACAAAATG | R: TGAGGTCAATGAAGGGGTCGT |

**Table S2.** Effects of CR on velocity and number of crossings of the central zone of mice in OFT.

| Group | Velocity (cm·s^-1^) | Central zone crossings (n) |
| --- | --- | --- |
| Con | 7.74 ± 0.29 | 59.30 ± 3.64 |
| Mod | 9.78 ± 0.42^#^ | 19.82 ± 2.67^###^ |
| Don | 9.36 ± 0.55 | 29.11 ± 3.01 |
| CR-L | 9.37 ± 0.59 | 23.33 ± 4.06 |
| CR-M | 8.07 ± 0.36^*^ | 44.10 ± 2.93^***^ |
| CR-H | 7.36 ± 0.48^**^ | 49.33 ± 3.13^***^ |

The date were presented as the mean ± *SEM*, *n* = 9 ~ 11; ^#^*P* < 0.05, ^##^*P* < 0.01, ^###^*P* < 0.001 vs. the Con group; ^*^*P* < 0.05, ^**^*P* < 0.01, ^***^*P* < 0.001, vs. the Mod group.

**Table S3.** Effects of CR on the recognition index of mice in NORT. (Left: the new object, Right: the old object).

| Group | recognition index (%) |
| --- | --- |
| Con | 67.78 ± 3.92 |
| Mod | 42.74 ± 4.15^#^ |
| Don | 70.32 ± 6.18^**^ |
| CR-L | 46.02 ± 4.22 |
| CR-M | 68.48 ± 3.80^*^ |
| CR-H | 70.38 ± 5.36^**^ |

The date were presented as the mean ± *SEM*, *n* = 9 ~ 11; ^#^*P* < 0.05, ^##^*P* < 0.01, ^###^*P* < 0.001 vs. the Con group; ^*^*P* < 0.05, ^**^*P* < 0.01, ^***^*P* < 0.001, vs. the Mod group.

**Table S4.** Effects of CR on the escape latency on the 4th day and number of platform crossings of mice in MWM.

| Group | escape latency on the 4th day (s) | platform crossings (n) |
| --- | --- | --- |
| Con | 22.36 ± 2.79 | 3.44 ± 0.29 |
| Mod | 47.94 ± 3.49^###^ | 1.29 ± 0.16^###^ |
| Don | 22.95 ± 2.40^***^ | 2.85 ± 0.32^***^ |
| CR-L | 36.52 ± 3.72^*^ | 1.19 ± 0.17 |
| CR-M | 26.13 ± 4.56^***^ | 2.68 ± 0.21^**^ |
| CR-H | 20.15 ± 2.32^***^ | 3.18 ± 0.35^***^ |

The date were presented as the mean ± *SEM*, *n* = 9 ~ 14; ^#^*P* < 0.05, ^##^*P* < 0.01, ^###^*P* < 0.001 vs. the Con group; ^*^*P* < 0.05, ^**^*P* < 0.01, ^***^*P* < 0.001, vs. the Mod group.

**Table S5.** Effect of CR on the levels of ACh and AChE in mice brain.

| Group | ACh (μg·mg^-1^) | AChE (nmol·g^-1^) |
| --- | --- | --- |
| Con | 333.2 ± 17.54 | 33.11 ± 1.674 |
| Mod | 191.7 ± 9.67^###^ | 53.42 ± 2.95^###^ |
| Don | 297.8 ± 16.96^***^ | 37.58 ± 3.65^***^ |
| CR-L | 224.2 ± 5.15 | 49.31 ± 3.07 |
| CR-M | 246.1 ± 8.77^*^ | 43.49 ± 2.35^*^ |
| CR-H | 277.9 ± 14.84^***^ | 32.51 ± 2.93^***^ |

The date were presented as the mean ± *SEM*, *n* = 7; ^#^*P* < 0.05, ^##^*P* < 0.01, ^###^*P* < 0.001 vs. the Con group; ^*^*P* < 0.05, ^**^*P* < 0.01, ^***^*P* < 0.001, vs. the Mod group.

**Table S6.** Effect of CR on the levels of IL-6, IL-1β, COX-2, TNF-α, iNOS and NO in mice brain.

| Group | IL-6  (pg·mg^-1^) | IL-1β  (pg·mg^-1^) | COX-2  (ng·mg^-1^) | TNF-α  (pg·mg^-1^) | iNOS  (ng·mg^-1^) | NO  (μmol) |
| --- | --- | --- | --- | --- | --- | --- |
| Con | 24.48 ± 1.20 | 22.75 ± 1.67 | 14.72 ± 0.84 | 138.3 ± 8.3 | 4.83 ± 0.32 | 12.24 ± 0.61 |
| Mod | 43.20 ± 2.47^###^ | 39.87 ± 1.33^###^ | 25.18 ± 0.78^###^ | 212.6 ± 11.79^###^ | 7.76 ± 0.44^##^ | 22.04 ± 1.07^###^ |
| Don | 26.55 ± 2.02^***^ | 25.50 ± 1.97^**^ | 16.88 ± 1.30^***^ | 136.3 ± 7.85^**^ | 5.49 ± 0.40^**^ | 16.40 ± 0.45^***^ |
| CR-L | 39.12 ± 1.99 | 38.78 ± 1.14 | 21.90 ± 0.91^*^ | 191.6 ± 11.34 | 7.18 ± 0.36 | 19.77 ± 0.87^*^ |
| CR-M | 33.05 ± 1.90^**^ | 32.10 ± 2.73^*^ | 20.21 ± 1.23^**^ | 178.0 ± 9.54^*^ | 6.91 ± 0.35 | 16.79 ± 0.49^**^ |
| CR-H | 23.38 ± 1.65^***^ | 25.86 ± 1.95^**^ | 16.27 ± 1.35^***^ | 151.3 ± 10.39^**^ | 5.77 ± 0.50^**^ | 15.08 ± 0.68^***^ |

The date were presented as the mean ± *SEM*, *n* = 7; ^#^*P* < 0.05, ^##^*P* < 0.01, ^###^*P* < 0.001 vs. the Con group; ^*^*P* < 0.05, ^**^*P* < 0.01, ^***^*P* < 0.001, vs. the Mod group.

**Table S7.** Effect of CR on the levels of MDA, SOD and GSH-Px in mice brain.

| Group | MDA (nmol·mg^-1^) | SOD (ng·mg^-1^) | GSH-Px (ng·mg^-1^) |
| --- | --- | --- | --- |
| Con | 4.91 ± 0.33 | 35.23 ± 2.24 | 68.37 ± 3.56 |
| Mod | 7.74 ± 0.28^##^ | 22.99 ± 1.87^##^ | 44.08 ± 3.44^###^ |
| Don | 4.59 ± 0.34^***^ | 33.27 ± 2.13^**^ | 57.21 ± 1.52^**^ |
| CR-L | 6.57 ± 0.35^*^ | 27.86 ± 1.78 | 50.22 ± 4.24 |
| CR-M | 6.07 ± 0.44^**^ | 32.37 ± 1.59^**^ | 59.35 ± 2.93^**^ |
| CR-H | 4.54 ± 0.21^***^ | 36.01 ± 2.00^***^ | 161.07 ± 3.38^***^ |

The date were presented as the mean ± *SEM*, *n* = 7; ^#^*P* < 0.05, ^##^*P* < 0.01, ^###^*P* < 0.001 vs. the Con group; ^*^*P* < 0.05, ^**^*P* < 0.01, ^***^*P* < 0.001, vs. the Mod group.

**Table S8.** Identification of alkaloids in the extract of CR and serum with drug

| No. | tR/min | Measured  m/z | Error  /ppm | Fragment  m/z | Molecular formula | Identification | Type | Ion form | Into the blood or not |
| --- | --- | --- | --- | --- | --- | --- | --- | --- | --- |
| 1 | 14.96 | 328.1574 | 9.4 | 328.1551，178.0869，163.0686，151.0802 | C_19_H_21_NO_4_ | Scoulerin | Protoberberine  alkaloid | [M+H]^+^ |  |
| 2 | 16.71 | 342.1712 | 3.4 | 192.1054，189.0787，177.0816 | C_20_H_23_NO_4_ | Norglaucine | Aporphine  alkaloid | [M+H]^+^ |  |
| 3 | 17.85 | 328.163 | 5.9 | 297.1188，265.0870，237.0964，191.0893 | C_19_H_21_NO_4_ | D-Isoboldine | Aporphine  alkaloid | [M+H]^+^ | √ |
| 4 | 18.04 | 342.1727 | 7.8 | 342.1762，192.1048，176.0764，148.0788 | C_20_H_23_NO_4_ | Lirioferine | Aporphine  alkaloid | [M+H]^+^ |  |
| 5 | 18.45 | 342.1725 | 7.3 | 192.1048，165.0935 | C_20_H_23_NO_4_ | Corypalmine | Protoberberine  alkaloid | [M+H]^+^ | √ |
| 6 | 18.84 | 342.1728 | 7.5 | 311.1305，296.1071，279.1038， | C_20_H_23_NO_4_ | Thaliporphine | Aporphine  alkaloid | [M+H]^+^ | √ |
| 7 | 19.62 | 368.1163 | 9.3 | 320.0950，292.1004，262.0900 | C_20_H_17_NO_6_ | (+)-Bicuculline | Others | [M+H]^+^ |  |
| 8 | 19.84 | 342.1728 | 7.2 | 342.1728，320.0950，178.0894，163.0659 | C_20_H_23_NO_4_ | Corydalmine | Protoberberine  alkaloid | [M+H]^+^ |  |
| 9 | 20.21 | 356.1879 | -1.4 | 356.1857，192.1048，178.0869，165.0766 | C_21_H_25_NO_4_ | Yuanhunine | Protoberberine  alkaloid | [M+H]^+^ | √ |
| 10 | 20.38 | 342.1728 | 7.6 | 342.1726，178.0869，163.0686 | C_20_H_23_NO_4_ | Tetrahydrocolumbamine | Protoberberine  alkaloid | [M+H]^+^ | √ |
| 11 | 20.75 | 356.1857 | 6.9 | 192.1048，176.0706，165.0935，148.0788 | C_21_H_25_NO_4_ | Coryphenanthrine | Others | [M+H]^+^ | √ |
| 12 | 20.9 | 324.12576 | -2.5 | 308.0953，292.0998，280.1009 | C_19_H_18_NO_4_^+^ | Demethyleneberberine | Protoberberine  alkaloid | [M]^+^ |  |
| 13 | 20.92 | 324.1258 | 5.6 | 324.1254，176.0764，149.0635 | C_19_H_17_NO_4_ | dl-Tetrahydrocoptisine | Protoberberine  alkaloid | [M+H]^+^ | √ |
| 14* | 21.13 | 354.1363 | 7.6 | 354.1364，190.0828，189.0789 | C_20_H_19_NO_5_ | Protopine | Protopine  alkaloid | [M+H]^+^ | √ |
| 15 | 21.63 | 340.1568 | -9.1 | 340.1568，292.1003，278.0856 | C_20_H_21_NO_4_ | Nantenine | Aporphine  alkaloid | [M+H]^+^ | √ |
| 16* | 21.79 | 356.1879 | -1.4 | 356.1857，294.1307，192.1048 | C_21_H_25_NO_4_ | Glaucine | Aporphine  alkaloid | [M+H]^+^ | √ |
| 17 | 22.36 | 370.1678 | -0.3 | 370.1653，206.0827，189.0785，188.0771 | C_21_H_23_NO_5_ | Allocryptopine | Protopine  alkaloid | [M+H]^+^ |  |
| 18 | 22.45 | 192.0648 | -3.6 | 189.0725，178.0811，176.0648 | C_10_H_9_NO_3_ | Noroxyhydrastinine | Others | [M+H]^+^ |  |
| 19 | 22.48 | 356.1939 | -5 | 311.09483，296.1071，279.0907 | C_21_H_26_NO_4_^+^ | Menisperine | Aporphine  alkaloid | [M]^+^ |  |
| 20* | 22.50 | 356.1881 | 5.8 | 356.1857，178.0869，192.1048，163.0686 | C_21_H_25_NO_4_ | Tetrahydropalmatine | Protoberberine  alkaloid | [M+H]^+^ | √ |
| 21 | 22.52 | 354.1712 | 3.5 | 325.1460，176.0730 | C_21_H_23_NO_4_ | Didehydroglaucine | Aporphine  alkaloid | [M+H]^+^ | √ |
| 22 | 22.68 | 328.1935 | 8.3 | 328.1946，283.1368，176.0764 | C_20_H_25_NO_3_ | Leonticine | Others | [M+H]^+^ |  |
| 23 | 22.70 | 325.1300 | 2.5 | 324.1257，308.0952，280.1009，266.0857 | C_19_H_18_NO_4_ | Demethyleneberberine | Protoberberine  alkaloid | [M+H]+ |  |
| 24 | 22.88 | 338.1413 | 2.4 | 338.1413，293.0706，264.0681 | C_20_H_19_NO_4_ | Tetrahydrocorysamine | Protoberberine  alkaloid | [M+H]^+^ | √ |
| 25* | 22.99 | 338.1413 | 6.5 | 338.1414，324.1254，280.1031，192.1048，176.0706 | C_20_H_20_NO_4_^+^ | Columbamine | Protoberberine  alkaloid | [M]^+^ |  |
| 26* | 23.15 | 320.0947 | 9.4 | 370.2074，320.0972，192.1048，176.0764，165.0935 | C_19_H_14_NO_4_^+^ | Coptisine | Protoberberine  alkaloid | [M]^+^ | √ |
| 27* | 23.65 | 340.1573 | 7.5 | 340.1599，192.1048，176.0764，165.0935 | C_20_H_21_NO_4_ | (R)-Canadine | Protoberberine  alkaloid | [M+H]^+^ | √ |
| 28* | 23.92 | 370.2034 | 6.8 | 370.2074，192.1048，176.0764 | C_22_H_27_NO_4_ | D-Corydaline | Protoberberine  alkaloid | [M+H]^+^ | √ |
| 29 | 24.38 | 336.125 | 8.1 | 336.1254，308.1226，294.1145 | C_19_H_13_NO_5_ | 8-Oxocoptisine | Protoberberine  alkaloid | [M+H]^+^ |  |
| 30 | 24.66 | 334.1101 | 8.3 | 334.1101，306.1143 | C_20_H_15_NO_4_ | Dihydrosanguinarine | Others | [M+H]^+^ | √ |
| 31 | 24.88 | 338.1410 | 6.7 | 338.1409，321.1019，176.0732 | C_20_H_19_NO_4_ | Dehydronanteine | Aporphine  alkaloid | [M+H]^+^ |  |
| 32* | 25.25 | 352.1567 | 3.7 | 352.1590，336.0969，320.0972，292.0674，278.0824 | C_21_H_22_NO_4_^+^ | Palmatine | Protoberberine  alkaloid | [M]+ | √ |
| 33* | 25.46 | 336.125 | 5.9 | 336.1289，320.0972，294.1157，292.0973 | C_20_H_18_NO_4_^+^ | Berberine | Protoberberine  alkaloid | [M]^+^ | √ |
| 34* | 26.16 | 366.1725 | -4.6 | 366.1688，350.1461，351.1518，308.1330，322.1473 | C_22_H_24_NO_4_^+^ | Dehydrocorydaline | Protoberberine  alkaloid | [M]^+^ | √ |
| 35 | 26.67 | 350.1412 | -3.7 | 350.1411，351.1451，334.1108 | C_21_H_19_NO_4_ | Dihydrochelerythrine | Others | [M+H]^+^ | √ |
| 36 | 26.92 | 334.1145 | 9.8 | 334.1145，306.1127，262.0882 | C_20_H_16_NO_4_^+^ | Worenine | Protoberberine  alkaloid | [M]^+^ |  |
| 37 | 33.18 | 368.1515 | 5.4 | 368.1517，324.1255，280.1003，266.0856，250.08849 | C_21_H_21_NO_5_ | Corynoline | Protoberberine  alkaloid | [M+H]^+^ | √ |
| 38 | 34.19 | 382.1319 | 6.5 | 382.1316，336.0889，308.0947，265.0799， | C_21_H_19_NO_6_ | Pontevedrine | Protoberberine  alkaloid | [M+H]^+^ |  |
| 39 | 40.41 | 338.1414 | 6.5 | 324.1254，308.1253，280.1031，266.0833 | C_20_H_20_NO_4_^+^ | Jatrorrhizine | Protoberberine  alkaloid | [M]+ | √ |
| 40 | 41.06 | 352.159 | 6.7 | 352.1590，320.0972，308.1330，294.1157，292.0973，248.0717 | C_21_H_22_NO_4_^+^ | Dehydrocorybulbine | Protoberberine  alkaloid | [M]+ | √ |
| 41 | 41.07 | 398.1628 | 7.5 | 398.1647，367.1387，352.1590，336.1289 | C_22_H_23_NO_6_ | Saulatine | Others | [M+H]^+^ |  |
| 42 | 42.26 | 370.1677 | 1.2 | 370.1676，352.1590，309.2091 | C_21_H_23_NO_5_ | Fumaricine | Others | [M+H]^+^ | √ |
| 43 | 44.84 | 352.1208 | -1.4 | 352.1180，337.0983，307.0838，235.0692 | C_20_H_17_NO_5_ | Oxoglaucine | Aporphine  alkaloid | [M+H]^+^ |  |

Note: * Compared with a standard substance; √ The prototype components of CR extract into the blood.

**Table S9.** The candidate bioactive components of CR.

| MOL ID | Compound name | OB (%) | DL |
| --- | --- | --- | --- |
| MOL001454 | Berberine | 36.86 | 0.78 |
| MOL001458 | Coptisine | 30.67 | 0.86 |
| MOL001461 | Dihydrochelerythrine | 32.73 | 0.81 |
| MOL001463 | Dihydrosanguinarine | 59.31 | 0.86 |
| MOL002903 | (R)-Canadine | 55.37 | 0.77 |
| MOL004071 | L-Tetrahydropalmatine | 73.94 | 0.64 |
| MOL004195 | Corydaline | 65.84 | 0.68 |
| MOL004198 | Corynoline | 46.06 | 0.85 |
| MOL004200 | Coryphenanthrine | 61.15 | 0.44 |
| MOL004203 | Dehydrocorybulbine | 46.97 | 0.63 |
| MOL004204 | Dehydrocorydaline | 41.98 | 0.68 |
| MOL004210 | fumaricine | 43.95 | 0.72 |
| MOL004763 | Izoteolin | 39.53 | 0.51 |
| MOL004226 | Protopine | 53.75 | 0.83 |
| MOL004230 | Stylopine | 48.25 | 0.85 |
| MOL004231 | Tetrahydrocorysamine | 34.17 | 0.86 |
| MOL000785 | Palmatine | 64.6 | 0.65 |
| MOL000790 | Isocorypalmine | 35.77 | 0.59 |

**Table S10.** The core targets of CR against AD in the PPI network.

| No. | Symbol | Name | Closeness | Degree | Betweenness |
| --- | --- | --- | --- | --- | --- |
| 1 | HSP90AA1 | Heat Shock Protein 90 Alpha Family Class A Member 1 | 0.523 | 104 | 0.110 |
| 2 | SRC | Proto-oncogene tyrosine-protein kinase Src | 0.533 | 100 | 0.112 |
| 3 | STAT3 | Signal Transducer And Activator Of Transcription 3 | 0.500 | 100 | 0.064 |
| 4 | EGFR | Epidermal Growth Factor Receptor | 0.514 | 88 | 0.052 |
| 5 | AKT1 | AKT Serine/Threonine Kinase 1 | 0.476 | 86 | 0.033 |
| 6 | MAPK1 | Mitogen-Activated Protein Kinase 1 | 0.498 | 76 | 0.049 |
| 7 | PIK3CA | Phosphatidylinositol-4,5-Bisphosphate 3-Kinase Catalytic Subunit Alpha | 0.447 | 74 | 0.016 |
| 8 | MAPK8 | Mitogen-Activated Protein Kinase 8 | 0.468 | 66 | 0.060 |
| 9 | CASP3 | Caspase 3 | 0.482 | 64 | 0.041 |
| 10 | IL6 | Interleukin 6 | 0.450 | 62 | 0.042 |
| 11 | TNF | Tumor Necrosis Factor | 0.443 | 56 | 0.039 |
| 12 | ESR1 | Estrogen Receptor 1 | 0.445 | 54 | 0.029 |
| 13 | NFKB1 | Nuclear Factor Kappa B Subunit 1 | 0.464 | 52 | 0.014 |
| 14 | JAK2 | Janus Kinase 2 | 0.438 | 52 | 0.009 |
| 15 | MAPK14 | Mitogen-Activated Protein Kinase 14 | 0.438 | 50 | 0.005 |
| 16 | CDC42 | Cell Division Cycle 42 | 0.438 | 48 | 0.006 |
| 17 | TLR4 | Toll Like Receptor 4 | 0.461 | 48 | 0.032 |
| 18 | RAC1 | Rac Family Small GTPase 1 | 0.443 | 46 | 0.008 |
| 19 | PIK3CD | Phosphatidylinositol-4,5-Bisphosphate 3-Kinase Catalytic Subunit Delta | 0.415 | 46 | 0.006 |
| 20 | PRKACA | Protein Kinase CAMP-Activated Catalytic Subunit Alpha | 0.429 | 46 | 0.039 |
| 21 | ERBB2 | Erb-B2 Receptor Tyrosine Kinase 2 | 0.445 | 44 | 0.018 |
| 22 | APP | Amyloid Beta Precursor Protein | 0.454 | 44 | 0.082 |
| 23 | IL1B | Interleukin 1 Beta | 0.426 | 44 | 0.012 |
| 24 | JAK1 | Janus Kinase 1 | 0.426 | 44 | 0.004 |
| 25 | BCL2L1 | BCL2 Like 1 | 0.454 | 42 | 0.022 |
| 26 | GSK3B | Glycogen Synthase Kinase 3 Beta | 0.445 | 42 | 0.014 |
| 27 | AKT2 | AKT Serine/Threonine Kinase 2 | 0.421 | 42 | 0.003 |
| 28 | MMP9 | Matrix Metallopeptidase 9 | 0.424 | 40 | 0.039 |
| 29 | MAP2K1 | Mitogen-Activated Protein Kinase Kinase 1 | 0.431 | 40 | 0.006 |
| 30 | MAPT | Microtubule Associated Protein Tau | 0.451 | 38 | 0.029 |
| 31 | MDM2 | MDM2 Proto-Oncogene | 0.429 | 36 | 0.015 |
| 32 | RPS6KB1 | Ribosomal Protein S6 Kinase B1 | 0.425 | 36 | 0.005 |
| 33 | RAF1 | Serine/Threonine Kinase | 0.426 | 36 | 0.003 |

**Table S11.** KEGG enrichment analysis of the putative targets of CR for AD treatment.

| NO. | term | -1og10(*P*) |
| --- | --- | --- |
| 1 | hsa05417:Lipid and atherosclerosis | 47.06 |
| 2 | hsa04151:PI3K-Akt signaling pathway | 36.96 |
| 3 | hsa04010:MAPK signaling pathway | 28.08 |
| 4 | hsa04066:HIF-1 signaling pathway | 23.28 |
| 5 | hsa04668:TNF signaling pathway | 22.88 |
| 6 | hsa04722:Neurotrophin signaling pathway | 20.89 |
| 7 | hsa04620:Toll-like receptor signaling pathway | 20.35 |
| 8 | hsa04915:Estrogen signaling pathway | 19.69 |
| 9 | hsa04630:JAK-STAT signaling pathway | 15.40 |
| 10 | hsa04150:mTOR signaling pathway | 10.50 |
| 11 | hsa05022:Pathways of neurodegeneration - multiple diseases | 29.56 |
| 12 | hsa04210:Apoptosis | 26.16 |
| 13 | hsa04024:cAMP signaling pathway | 25.49 |
| 14 | hsa04725:Cholinergic synapse | 16.58 |
| 15 | hsa04020:Calcium signaling pathway | 24.78 |
| 16 | hsa04080:Neuroactive ligand-receptor interaction | 17.78 |
| 17 | hsa04726:Serotonergic synapse | 22.81 |
| 18 | hsa04723:Retrograde endocannabinoid signaling | 9.50 |
| 19 | hsa04724:Glutamatergic synapse | 5.68 |
| 20 | hsa04064:NF-kappa B signaling pathway | 15.60 |

**Table S12.** Effect of CR on the levels of inflammatory mediators in the supernatant of LPS-induced BV2 cells.

| Group | Cell viability  (% con) | NO  (μM) | TNF-α  (pg·mL^-1^) | IL-1β  (pg·mL^-1^) | IL-6  (pg·mL^-1^) | IL-10  (pg·mL^-1^) |
| --- | --- | --- | --- | --- | --- | --- |
| Con | 100 | 1.08 ± 0.12 | 279.8 ± 8.54 | 63.45 ± 2.10 | 56.78 ± 3.49 | 50.52 ± 2.64 |
| Mod | 104.5 ± 1.09 | 25.80 ± 0.90^###^ | 557.2 ± 16.73^###^ | 126.6 ± 5.05^###^ | 106.9 ± 3.19^###^ | 64.23 ± 3.07 |
| Don | 115.7 ± 1.46 | 9.75 ± 0.83^***^ | 333.3 ± 21.54^***^ | 83.76 ± 4.18^***^ | 58.42 ± 3.69^***^ | 93.50 ± 3.73^***^ |
| 10 | 108.9 ± 1.30 | 14.70 ± 0.31^***^ | 422.0 ± 41.61^*^ | 117.0 ± 2.61 | 84.19 ± 2.98^**^ | 72.89 ± 2.41 |
| 20 | 108.4 ± 1.11 | 6.80 ± 0.25^***^ | 292.8 ± 22.04^***^ | 84.21 ± 3.49^***^ | 60.38 ± 5.74^***^ | 115.8 ± 4.19^***^ |

The date were presented as the mean ± *SEM*, *n* = 5; ^#^*P* < 0.05, ^##^*P* < 0.01, ^###^*P* < 0.001 vs. the Con group; ^*^*P* < 0.05, ^**^*P* < 0.01, ^***^*P* < 0.001, vs. the Mod group.

**Table S13.** Effect of CR on p-JAK2/JAK2 and p-STAT3/STAT3 ratios in BV2 cells.

| Group | p-JAK2/JAK2 | p-STAT3/STAT3 |
| --- | --- | --- |
| Con | 0.81 ± 0.06 | 0.74 ± 0.02 |
| Mod | 2.00 ± 0.06^###^ | 1.52 ± 0.08^###^ |
| Don | 1.57 ± 0.05^*^ | 1.11 ± 0.06^**^ |
| 10 | 1.83 ± 0.11 | 1.33 ± 0.02 |
| 20 | 1.39 ± 0.12^**^ | 1.02 ± 0.01^***^ |

The date were presented as the mean ± *SEM*, *n* =3; ^#^*P* < 0.05, ^##^*P* < 0.01, ^###^*P* < 0.001 vs. the Con group; ^*^*P* < 0.05, ^**^*P* < 0.01, ^***^*P* < 0.001, vs. the Mod group.

**Table S14.** Effect of CR on M1 marker (CD16) and M2 marker (CD206) mRNA expression in BV2 cells.

| Group | relative mRNA expression of CD16 | relative mRNA expression of CD206 |
| --- | --- | --- |
| Con | 1.02 ± 0.01 | 0.98 ± 0.02 |
| Mod | 2.05 ± 0.02^###^ | 0.89 ± 0.01 |
| Don | 1.57 ± 0.02^**^ | 1.55 ± 0.01^*^ |
| 10 | 1.74 ± 0.01^*^ | 1.19 ± 0.01 |
| 20 | 1.45 ± 0.01^**^ | 1.73 ± 0.02^**^ |

The date were presented as the mean ± *SEM*, *n* =3; ^#^*P* < 0.05, ^##^*P* < 0.01, ^###^*P* < 0.001 vs. the Con group; ^*^*P* < 0.05, ^**^*P* < 0.01, ^***^*P* < 0.001, vs. the Mod group.
